# Acute Diabetes Complications After Transition to a Value-Based Medication Benefit

**DOI:** 10.1001/jamahealthforum.2023.5309

**Published:** 2024-02-09

**Authors:** J. Franklin Wharam, Stephanie Argetsinger, Matthew Lakoma, Fang Zhang, Dennis Ross-Degnan

**Affiliations:** 1 Department of Medicine, Duke University, Durham, North Carolina; 2 Duke-Margolis Center for Health Policy, Durham, North Carolina; 3 Department of Population Medicine, Harvard Pilgrim Health Care Institute and Harvard Medical School, Boston, Massachusetts

## Abstract

**Importance:**

The association of value-based medication benefits with diabetes health outcomes is uncertain.

**Objective:**

To assess the association of a preventive drug list (PDL) value-based medication benefit with acute, preventable diabetes complications.

**Design, Setting, and Participants:**

This cohort study used a controlled interrupted time series design and analyzed data from a large, national, commercial health plan from January 1, 2004, through June 30, 2017, for patients with diabetes aged 12 to 64 years enrolled through employers that adopted PDLs (intervention group) and matched and weighted members with diabetes whose employers did not adopt PDLs (control group). All participants were continuously enrolled and analyzed for 1 year before and after the index date. Subgroup analysis assessed patients with diabetes living in lower-income and higher-income neighborhoods. Data analysis was performed between August 19, 2020, and December 1, 2023.

**Exposure:**

At the index date, intervention group members experienced employer-mandated enrollment in a PDL benefit that was added to their follow-up year health plan. This benefit reduced out-of-pocket costs for common cardiometabolic drugs, including noninsulin antidiabetic agents and insulin. Matched control group members continued to have cardiometabolic medications subject to deductibles or co-payments at follow-up.

**Main Outcomes and Measures:**

The primary outcome was acute, preventable diabetes complications (eg, bacterial infections, neurovascular events, acute coronary disease, and diabetic ketoacidosis) measured as complication days per 1000 members per year. Intermediate measures included the proportion of days covered by and higher use (mean of 1 or more 30-day fills per month) of antidiabetic agents.

**Results:**

The study 10 588 patients in the intervention group (55.2% male; mean [SD] age, 51.1 [10.1] years) and 690 075 patients in the control group (55.2% male; mean [SD] age, 51.1 [10.1] years) after matching and weighting. From baseline to follow-up, the proportion of days covered by noninsulin antidiabetic agents increased by 4.7% (95% CI, 3.2%-6.2%) in the PDL group and by 7.3% (95% CI, 5.1%-9.5%) among PDL members from lower-income areas compared with controls. Higher use of noninsulin antidiabetic agents increased by 11.3% (95% CI, 8.2%-14.5%) in the PDL group and by 15.2% (95% CI, 10.6%-19.8%) among members of the PDL group from lower-income areas compared with controls. The PDL group experienced an 8.4% relative reduction in complication days (95% CI, −13.9% to −2.8%; absolute reduction, −20.2 [95% CI, −34.3 to −6.2] per 1000 members per year) compared with controls from baseline to follow-up, while PDL members residing in lower-income areas had a 10.2% relative reduction (95% CI, −17.4% to −3.0%; absolute, −26.1 [95% CI, −45.8 to −6.5] per 1000 members per year).

**Conclusions and Relevance:**

In this cohort study, acute, preventable diabetes complication days decreased by 8.4% in the overall PDL group and by 10.2% among PDL members from lower-income areas compared with the control group. The results may support a strategy of incentivizing adoption of targeted cost-sharing reductions among commercially insured patients with diabetes and lower income to enhance health outcomes.

## Key Points

**Question:**

Are value-based insurance designs that lower out-of-pocket costs for cardiometabolic medications, including antidiabetic agents, associated with improved short-term health among patients with diabetes?

**Findings:**

In this cohort study of 10 588 patients with diabetes who were switched to a value-based medication benefit and 690 075 matched and weighted control individuals, the intervention group experienced an 8% reduction in acute, preventable diabetes complication days, with a corresponding 10% decline among those residing in lower-income areas.

**Meaning:**

Reducing out-of-pocket costs for cardiometabolic medications may be associated with modestly improved short-term health outcomes among commercially insured patients with diabetes.

## Teaser

This cohort study examines the association between transition to valued-based preventive drug list benefits and acute, preventable diabetes complications.

## Introduction

Type 2 diabetes is a complex disease characterized by insulin resistance, relative insulin insufficiency, and impaired glucose, lipid, and protein metabolism.^1^ Prevalence has increased from approximately 10% to 13% in the past 20 years.^2^ Pharmacotherapy with antidiabetic agents, antihypertensives, and lipid-lowering drugs is an essential component of secondary prevention of diabetes complications.^1^

Acute and chronic diabetes complications are rising among working-age people,^3,4^ but causes remain uncertain. Experts have suggested factors such as increasing morbidity among younger, socioeconomically disadvantaged populations; stagnating preventive care; and out-of-pocket cost barriers.^4^ Out-of-pocket costs continue to grow in commercial health benefits.^5^ Nationally, patients with diabetes pay approximately $150 to $500 per year for antidiabetic medications.^6^ Most employer-sponsored plans, whether or not they have a high deductible for medical services, apply co-payments to prescriptions.^7^ A less common plan type,^8^ high-deductible health plans with health savings accounts, subjects almost all medications to the deductible.^9^

In response to concerns about out-of-pocket cost barriers,^10,11,12^ some employers have adopted more generous health insurance designs over the past decade.^13,14,15^ Employers can purchase a preventive drug list (PDL) add-on, allowing enrollees to pay reduced out-of-pocket co-payments or deductible amounts for a range of high-value preventive medications, including antidiabetic drugs, antihypertensives, and lipid-lowering agents.^16^

Recent evidence has demonstrated that PDLs are associated with increased preventive medication fills (including antidiabetic agents, antihypertensives, lipid-lowering agents, and asthma medications) among patients with diabetes from lower-income neighborhoods.^16^ However, research examining associations of value-based medication benefits with important health outcomes has been limited^17,18^ and has not assessed the association with lower income status or diabetes clinical end points.

We therefore studied whether PDLs are associated with reduced rates of acute, preventable diabetes complications. We studied a 14-year rolling cohort (2004-2017) in a dataset that includes approximately 2.3 million commercially insured patients with diabetes. We hypothesized that transitions to more generous medication coverage would be associated with reduced acute, preventable diabetes complication days. We expected these outcomes to be more pronounced among PDL members from lower-income vs higher-income neighborhoods.

## Methods

### Study Population

In this cohort study using a controlled interrupted time series design, we collected data from commercially insured people with diabetes in a large national commercial (and Medicare Advantage) claims database between January 1, 2004, and June 30, 2017; we excluded members with Medicare coverage. We performed the data analysis between August 19, 2020, and December 1, 2023. The Duke University and Harvard Pilgrim Health Care institutional review boards approved this study and waived informed consent because deidentified data were used. This study followed the Strengthening the Reporting of Observational Studies in Epidemiology (STROBE) reporting guideline.

Data elements included details on enrollment, deductible level, health savings account eligibility, census tract of residence, and all medical and pharmacy claims. The PDL plans offered by the national insurer in our data are of 2 basic types: core and expanded. Diabetes medicines and supplies are included only on the expanded list, while antihypertensives and lipid-lowering agents are on both lists (eTables 1 and 2 in Supplement 1). We had no direct measure of employers’ PDL status per benefit year, so we imputed their presence^16^ (eMethods in Supplement 1). We first identified all products on the PDLs using the National Drug Data File Plus (First DataBank, Inc) and then extracted pharmacy claims for all members in every account. We captured all out-of-pocket (deductible or co-payment) amounts per claim paid for PDL and non-PDL medications. Using rules based on the percentages of claims with deductible payments and co-payments for PDL and non-PDL medications (eMethods in Supplement 1), we identified employer account months and benefit years with patterns indicating PDL adoption. For example, using the first 6 months of a benefit year, if more than 90% of claims in a month for PDL-listed medications had no co-payment or deductible payment while 30% or more of non-PDL medications had positive co-payments or deductible amounts, the month was assigned as a potential PDL benefit month. If an employer account met this criterion for at least 5 of the first 6 months of its benefit year, it was considered to have a PDL in that year. We also used 4 other rules based on full employer account years to flag PDL years. These included, for example, (1) more than 90% of fills for PDL-listed medications in the year with a deductible or co-pay of $0, and 30% or more of fills for non-PDL agents with a deductible or co-payment of more than $0 and (2) 0 fills in the year for PDL medications with a deductible or co-payment of more than $0 and 5 or more fills for non-PDL agents with a deductible or co-payment of more than $0. eTable 3 in Supplement 1 lists the full set of rules.

Most employers have only a single account or plan type with the health insurer represented in our data; if this account met 1 of the aforementioned rules in a given benefit year, we flagged it as a PDL employer year. If a single-account employer year met none of the PDL imputation rules, we flagged it as non-PDL. Other employers offer multiple benefit types through multiple accounts with the health insurer. In these cases, we pooled all accounts per employer and their nested enrollees. If at least 85% of enrollees at the employer in the benefit year were imputed to have PDLs, we considered the employer to have fully covered its enrollees with PDL access.

We classified multiple account employer years that had less than 15% of enrollees with PDL access as non-PDL. We required employers to have at least 2 consecutive benefit years. From these employers, we assigned members who were in a PDL after at least 1 year without a PDL to the intervention group pool. We assigned members from employers that never offered a PDL to the control group pool. In the intervention group pool, we defined the day of PDL benefit adoption as the index date and assigned contemporaneous index dates to control pool members (eMethods in Supplement 1). To minimize selection bias, we excluded members from employers that we classified as allowing enrollees a choice between joining and not joining a PDL.

We then identified all patients with diabetes aged 12 to 64 years using a standard claims-based algorithm (eTable 4 in Supplement 1). To be eligible, we required members to have been diagnosed with diabetes prior to the index date and to be continuously enrolled for 12 months before and after the index date.

We therefore created a cohort of intervention group members who did not have a PDL for a full baseline year and were then required to adopt an expanded PDL for a full follow-up year. The control pool had corresponding enrollment through employers that did not offer a PDL for 2 consecutive years.

### Matching and Matching Variables

To further minimize selection effects, we used the kmatch procedure in Stata, version 17 (StataCorp LLC)^19^ to balance multiple employer- and member-level characteristics. Stata kmatch allows combining exact matching on high-priority characteristics with close matching of other characteristics through entropy balancing^20,21^ using a propensity score to weight the control group within exact-match strata. We exact matched on category of census tract income level, whether members had high-deductible health plans with health savings accounts at baseline and follow-up vs traditional tiered drug co-payment plans at baseline and follow-up, and quarterly trends in category of baseline acute, preventable complication days (0, 1-4, and ≥5 days per quarter) (eMethods in Supplement 1). We included this baseline outcome trend term in the match based on evidence from rigorous within-study comparison studies^22^ of reduced bias in observational studies that include self-selected entities (employers in this case) and an uncertain functional form of the baseline trend. We defined lower income based on residence in census tracts with 10% or more of households below the federal poverty level.^23,24^

We included all other characteristics (eMethods in Supplement 1) in the kmatch propensity score calculation. Member-level characteristics included age group; sex; race and ethnicity; US region of residence; Johns Hopkins ACG morbidity score quartile^25^; baseline quarter of first fill for noninsulin antidiabetic, insulin, antihypertensive, and lipid-lowering agents, if any; decile of baseline out-of-pocket and total medical and pharmacy costs; baseline and follow-up health insurance provider network (broad vs narrow); and baseline and follow-up deductible level. Race and ethnicity were determined based on combining the 2010-2014 American Community Survey^26^ census tract level predominant race and ethnicity (Black, Hispanic, and White) with a superseding full-name analysis (Asian, Hispanic) provided through the data vendor (eMethods in Supplement 1); other includes people residing in census tracts that have less than 75% of residents who are Black, less than 75% of residents who are Hispanic, less than 75% of residents who are White, and participants not classified as having an Asian or a Hispanic name. We included race and ethnicity as a matching variable because diabetes control and complications are known to differ substantially across categories.^27^ Employer-level characteristics included year period (2004-2007, 2008-2009, 2010-2011, 2012, 2013, 2014, 2015, and 2016) and calendar quarter of index date; size category; proportion in age category; proportion female; proportion of members in census tract poverty category (below-poverty levels of <5%, 5%-9.9%, 10%-19.9%, and ≥20%); and decile of baseline out-of-pocket and total medical and pharmacy costs per enrollee per month.

### Measures of Exposure and Mediator

To assess the degree of relevant medication cost-sharing changes, we measured out-of-pocket costs per 30-day equivalent fill of insulin and noninsulin antidiabetic agents (including oral agents and injectables, such as semaglutide). We also created intermediate measures to assess whether the PDL was associated with meaningful improvements in antidiabetic medication use. We calculated percentage of days covered (PDC) by allocating the days’ supply for dispensed insulin and noninsulin antidiabetic agents over time and accounting for fills that overlap. This measure reflects consistent medication availability. We assessed the number of 30-day equivalent noninsulin antidiabetic medication fills and insulin fills per member per study period. In addition, we defined higher use of antidiabetic agents as a mean of at least one 30-day fill per month over the study period (eMethods in Supplement 1). We used these individual measures to calculate baseline and follow-up population mean PDC per period, mean antidiabetic 30-day equivalent fills per period, and percentages with higher use per period.

### Primary Outcome

We sought to measure diabetes health outcomes that could be improved with increased use of cardioprotective medications, especially antidiabetic agents. Such medications lower blood glucose levels, reducing the risk of acute infections and adverse effects of pronounced hyperglycemia. Other cardioprotective medications can reduce the risk of less common acute complications, such as severe hypertension, myocardial infarction, and stroke. We therefore used a previously created measure of acute, preventable diabetes complications,^12^ defined as diabetes-related symptoms or conditions associated with gaps in recommended care and medication use that require timely intervention by medical professionals (the eMethods in Supplement 1 gives the full definition). This measure was initially generated from an American Diabetes Association list of diabetes complications^28^ and the Agency for Healthcare Research and Quality’s claims-based Prevention Quality Indicators.^29^ Two clinicians (J.F.W. and a nonauthor clinician) masked to study group used a 5-question decision algorithm^12^ to independently classify conditions on the initial list as acute, preventable diabetes, followed by reconciliation of differences in classification.

A previous study by some of us^12^ validated this measure by determining that gaps in care were associated with increased complication rates and that outpatient and emergency department (ED) visits with these complication diagnoses were associated with a higher odds of subsequent hospitalization compared with other types of outpatient or ED visits (odds ratio, 4.10 [95% CI, 3.98-4.23] and 3.02 [95% CI, 2.96-3.08]), respectively). For the current study, we updated the measure to *International Statistical Classification of Diseases and Related Health Problems, Tenth Revision* codes by using the General Equivalence Mappings approach.^30^ An internal medicine clinician (J.F.W.) then applied measure criteria (eFigure in Supplement 1) to each candidate code to flag those that represented acute, preventable diabetes complications in either the outpatient (telehealth or office visit) or the high-acuity (ED, observation stay, or inpatient) setting (eMethods in Supplement 1). The measure was setting specific such that diagnoses might represent a complication in the high-acuity setting but not the outpatient setting. For example, we coded congestive heart failure as an acute complication only if generated in the ED or hospital given that office visits for this condition often represent routine monitoring rather than complications. eTable 5 in Supplement 1 includes the frequency of complication types at baseline; the top 10 most common (accounting for 92% of all baseline complications) were cellulitis, abscess, and related infections; urinary tract infections; serious respiratory tract infections; stroke or transient neurologic deficit; myocardial infarction or transient coronary artery ischemia; abnormal blood glucose level or closely related metabolic abnormalities; other infections; otitis externa; bacterial upper respiratory tract infections; and acute kidney failure.

Our measure of interest was the number of days presenting to the health system for a given complication subtype (eMethods in Supplement 1). For example, an ED visit for diabetic ketoacidosis on day 1 followed by a 3-day hospitalization would represent 4 acute complication days. We chose this measure because days lost to or affected by preventable health care utilization are important to both patients and employers, the purchaser of PDL benefits. Although this is a novel measure, it is conceptually similar to capturing morbidity and acuity using hospitalization days, a long-established proxy outcome in health systems research.^31^ We assigned 1 complication day per telehealth or office visit. We assigned the full duration of ED visits and observation stays and winsorized at 3 days. Similarly, we included each hospitalization day and winsorized at 7 days to minimize the influence of outliers.

### Statistical Analysis

We compared baseline characteristics of the study groups using a standardized differences approach.^32^ We determined that there were no baseline monthly trend differences in our primary outcome using segmented regression analysis with an autoregressive model^33^ (eMethods in Supplement 1). To visualize potential outcomes of PDL adoption, we plotted monthly weighted baseline and follow-up trends in cumulative complication days by study group.^34^

To generate intuitive relative and absolute change estimates of our intermediate and primary measures, we used marginal effects methods^35^ after running difference-in-differences models with generalized estimating equations.^36,37^ Models were at the annual level and used a binomial distribution for measures of PDC and higher antidiabetic medication use and a negative binomial distribution for measures of antidiabetic out-of-pocket costs per fill; 30-day equivalent antidiabetic medication fills; and acute, preventable complication days. Models took the form of *Y_i,t_* = β_0_ + β_1_study group*_i,t_* + β_2_study period*_i,t_* + β_3_study group*_i,t_* × study period*_i,t_* + ϵ*_i,t_*. *Y_i,t_* represented the outcome value of study participant *i* at period *t.* Study period was defined as baseline (0) or follow-up (1), and study group was defined as PDL (1) or control (0). The error term was ϵ*_i,t_*. The parameter estimate of interest, β_3_, represented the interaction between study group and study period. We adjusted all analyses with weights generated by the match and, thus, did not add other adjusting covariates to the model because they were already balanced by the weighting.

Given our hypothesis that PDL members with lower incomes would experience greater reductions in acute, preventable complication days than counterparts with higher incomes, we stratified all analyses into members residing in lower- vs higher-income areas. We used the same analytic approach for these subgroups as for the overall cohort. The statistical analysis was performed using Stata, versions 15.1 and 18. Results are reported based on 2-sided tests of statistical significance, defined as *P* < .05.

## Results

The final PDL cohort included 10 588 PDL members with diabetes (Table 1) and 1:65 matched, weighted non-PDL members (n = 690 075) (control group). Measured baseline characteristics of the intervention and control group were nearly identical after weighting. A total of 44.8% of members in both groups were female, 55.2% were male, 44.5% were aged 55 to 64 years (mean [SD] age, 51.1 [10.1] years), and 53.1% lived in lower-income areas. A total of 4.2% were classified as Asian; 4.3% lived in predominantly Black neighborhoods; 12.8% were classified as Hispanic; 54.6% lived in predominantly White neighborhoods; and 24.1% did not fall into these categories. Also, 45.5% were from the US South, and 33.4% had employers with fewer than 100 enrollees. Percentages of members with baseline year deductibles of $0 to $999, $1000 to $2499, and $2500 or more were 28.8%, 47.5%, and 23.5%, respectively. Baseline trends in acute, preventable diabetes complication days did not differ between the study groups (eTable 6 in Supplement 1).

**Table 1.** Baseline Characteristics of the 2 Study Cohorts Before and After Matching and Weighting

Characteristic	Participants, No. (%)			
	Before matching and weighting		After matching and weighting^a^	
	PDL group (n = 10 625)	Control pool (n = 695 998)	PDL group (n = 10 588)	Control group (n = 690 075)^b^
Sex				
Female	4762 (44.8)	323 574 (46.5)	4745 (44.8)	309 256 (44.8)
Male	5863 (55.2)	372 424 (53.5)	5843 (55.2)	380 819 (55.2)
Age at index date, y				
12-29	445 (4.2)	27 733 (4.0)	439 (4.1)	28 612 (4.1)
30-44	1853 (17.4)	128 029 (18.4)	1845 (17.4)	120 248 (17.4)
45-49	1534 (14.4)	93 875 (13.5)	1532 (14.5)	99 848 (14.5)
50-54	2068 (19.5)	128 410 (18.5)	2064 (19.5)	134 522 (19.5)
55-59	2412 (22.7)	154 120 (22.1)	2402 (22.7)	156 551 (22.7)
60-64	2313 (21.8)	163 831 (23.5)	2306 (21.8)	150 294 (21.8)
Lower income^c^	5635 (53.0)	399 898 (57.5)	5617 (53.1)	366 089 (53.1)
Race and ethnicity^d^				
Asian	448 (4.2)	26 794 (3.9)	447 (4.2)	29 133 (4.2)
Black	459 (4.3)	25 323 (3.6)	451 (4.3)	29 394 (4.3)
Hispanic	1364 (12.8)	92 700 (13.3)	1361 (12.8)	88 703 (12.8)
White	5796 (54.6)	369 663 (53.1)	5781 (54.6)	376 778 (54.6)
Other^e^	2558 (24.1)	181 518 (26.1)	2548 (24.1)	166 066 (24.1)
Region				
South	4828 (45.4)	358 931 (51.6)	4814 (45.5)	313 753 (45.5)
West	1858 (17.5)	109 083 (15.7)	1852 (17.5)	120 705 (17.5)
Northeast	639 (6.0)	47 767 (6.9)	635 (6.0)	41 386 (6.0)
Midwest	3300 (31.1)	180 217 (25.9)	3287 (31.0)	21 4231 (31.0)
Comorbidity score quartile^f^				
Low	3022 (28.4)	173 459 (24.9)	3014 (28.5)	196 438 (28.5)
Medium	2633 (24.8)	174 201 (25.0)	2627 (24.8)	171 215 (24.8)
High	2538 (23.9)	174 057 (25.0)	2530 (23.9)	164 893 (23.9)
Very high	2432 (22.9)	174 281 (25.0)	2417 (22.8)	157 529 (22.8)
Deductible level, $				
0-999	3062 (28.8)	464 275 (66.7)	3062 (28.8)	199 045 (28.8)
1000-2499	5055 (47.6)	174 252 (25.0)	5030 (47.5)	327 831 (47.5)
≥2500	2503 (23.6)	55 919 (8.0)	2491 (23.5)	162 351 (23.5)
Choice	5 (0.1)	1552 (0.2)	5 (0.1)	847 (0.1)
Employer size, No. of enrollees				
0-99	3544 (33.4)	201 951 (29.0)	3534 (33.4)	230 329 (33.4)
100-999	2245 (21.1)	198 759 (28.6)	2244 (21.2)	376 582 (21.2)
1000-4999	1514 (14.3)	122 598 (17.6)	1512 (14.3)	475 127 (14.3)
≥5000	3322 (31.3)	172 690 (24.8)	3298 (31.1)	690 075 (31.1)

Abbreviation: PDL, preventive drug list.

^a^ Characteristics of PDL (intervention) and control group members were nearly identical due to the weighting process; similarly, all standardized differences were less than 0.0001 (values closer to 0 indicate greater similarity).

^b^ Control group members were weighted, but the control group retained the sample size of members who had a weight greater than 0.

^c^ Residence in census tracts with 10% or more of households below the federal poverty level based on the 2010-2014 American Community Survey.^26^

^d^ Based on combining the 2010-2014 American Community Survey^26^ census tract–level predominant race and ethnicity (Black, Hispanic, and White) with a superseding full-name analysis (Asian, Hispanic) via the data vendor (eMethods in Supplement 1).

^e^ Other comprises people not included in the Asian, Black, Hispanic, and White categories (eMethods in Supplement 1).

^f^ Based on Johns Hopkins ACG software.^25^

### Measures of Exposure and Mediator

From baseline to follow-up, out-of-pocket costs per 30-day equivalent noninsulin antidiabetic agent and insulin declined by 30.7% (95% CI −32.6% to −28.8%) and 38.6% (95% CI, −41.1% to −36.2%), respectively, in the PDL group vs controls (Table 2). Reductions were similar among PDL members living in higher- and lower-income areas.

**Table 2.** Changes in Noninsulin Antidiabetic Medication and Insulin OOP Costs per Fill From Baseline to Follow-Up in the PDL Group vs Control Group^a^

Variable	Mean OOP cost, US $				Change in OOP cost^b^	
	PDL group		Control group			
	Baseline	Follow-Up	Baseline	Follow-Up	Absolute, $ (95% CI)	Relative, % (95% CI)
Overall cohort						
Noninsulin antidiabetics	18.1	13.2	18.6	19.6	−5.8 (−6.3 to −5.4)	−30.7 (−32.6 to −28.8)
Insulin	59.4	38.3	55.5	58.3	−24.1 (−26.3 to −21.9)	−38.6 (−41.1 to −36.2)
Higher-income group^c^						
Noninsulin antidiabetics	19.0	14.2	19.9	20.6	−5.4 (−6.1 to −4.7)	−27.7 (−30.6 to −24.7)
Insulin	58.9	38.5	54.6	57.8	−23.9 (−26.8 to −20.9)	−38.3 (−41.6 to −35.0)
Lower-income group^d^						
Noninsulin antidiabetics	17.3	12.3	17.5	18.7	−6.2 (−6.8 to −5.6)	−33.4 (−35.8 to −31.0)
Insulin	59.9	38.0	56.4	58.8	−24.4 (−27.6 to −21.2)	−39.1 (−42.6 to −35.5)

Abbreviations: OOP, out-of-pocket; PDL, preventive drug list.

^a^ Annual means and absolute and relative difference estimates were derived from difference-in-differences generalized estimating equations regression models with a negative binomial distribution weighted for matching covariates.

^b^ *P* < .05 for all.

^c^ Residence in census tracts with less than 10% of households below the federal poverty level based on the 2010-2014 American Community Survey.^26^

^d^ Residence in census tracts with 10% or more of households below the federal poverty level based on the 2010-2014 American Community Survey.^26^

The PDL group experienced a 7.1% (95% CI, 5.0%-9.3%) increase in noninsulin antidiabetic medication 30-day fills from baseline to follow-up compared with controls and a corresponding 5.3% (95% CI, 2.2%-8.4%) increase in insulin 30-day equivalent fills (Table 3). From baseline to follow-up, the PDC by noninsulin antidiabetic agents increased by 4.7% (95% CI, 3.2%-6.2%) in the overall PDL group compared with the control groups, while the respective increase for insulin was 3.1% (95% CI, 0.4%-5.9%). The proportion of members who had higher use of noninsulin antidiabetic agents increased by 11.3% (95% CI, 8.2%-14.5%) in the overall PDL group compared with controls, while the change for insulin in the overall PDL group (5.0%; 95% CI, −1.6% to 11.7%) was not statistically significant.

**Table 3.** Changes in Noninsulin Antidiabetic and Insulin Use in the PDL Group vs Control Group From Baseline to Follow-Up^a^

Variable	PDL group		Control group		Change in estimate	
	Baseline	Follow-Up	Baseline	Follow-Up	Absolute (95% CI)	Relative, % (95% CI)
**Overall cohort**						
Noninsulin antidiabetics						
PDC	44.5	44.5	43.8	41.8	2.0 (1.4 to 2.6)^b^	4.7 (3.2 to 6.2)^b^
30-d Fills per member per mo, No.^c^	0.57	0.66	0.57	0.62	0.04 (0.0 to 0.1)^b^	7.1 (5.0 to 9.3)^b^
Higher use, %^c^^,^^d^	25.2	30.5	26.1	28.3	3.1 (2.3 to 3.9)^b^	11.3 (8.2 to 14.5)^b^
Insulin						
PDC	14.3	15.5	13.8	14.4	0.5 (0.1 to 0.9)^b^	3.1 (0.4 to 5.9)^b^
30-d Fills per member per mo, No.^c^	0.17	0.19	0.17	0.18	0.01 (0.0 to 0.0)^b^	5.3 (2.2 to 8.4)^b^
Higher use, %^c^^,^^d^	6.6	8.0	6.5	7.4	0.4 (−0.1 to 0.9)	5.0 (−1.6 to 11.7)
**Higher-income group** ^e^						
Noninsulin antidiabetics						
PDC	45.0	44.2	44.0	42.4	0.8 (−0.1 to 1.7)	1.9 (−0.2 to 4.0)
30-d Fills per member per month, No.^c^	0.58	0.66	0.59	0.64	0.02 (0.0 to 0.0)^b^	3.5 (0.6 to 6.5)^b^
Higher use, %^c^^,^^d^	26.5	31.2	27.1	29.7	2.2 (1.0 to 3.3)^b^	7.4 (3.2 to 11.7)^b^
Insulin						
PDC	15.6	16.9	15.4	16.1	0.6 (0.0 to 1.2)^b^	3.7 (0.0 to 7.3)^b^
30-d Fills per member per month, No.^c^	0.19	0.22	0.19	0.21	0.01 (0.0 to 0.0)	3.8 (−0.3 to 7.8)
Higher use, %^c^^,^^d^	7.9	9.3	7.7	9.1	0.0 (−0.7 to 0.8)	0.4 (−8.0 to 8.8)
**Lower-income group** ^f^						
Noninsulin antidiabetics						
PDC	44.1	44.7	43.6	41.2	3.1 (2.2 to 3.9)^b^	7.3 (5.1 to 9.5)^b^
30-d Fills per member per month, No.^c^	0.55	0.66	0.56	0.61	0.06 (0.0 to 0.1)^b^	10.5 (7.4 to 13.6)^b^
Higher use, %^c^^,^^d^	24.1	29.9	25.1	27.1	3.9 (2.8 to 5.1)^b^	15.2 (10.6 to 19.8)^b^
Insulin						
PDC	13.2	14.2	12.3	12.9	0.4 (−0.2 to 0.9)	2.5 (−1.6 to 6.7)
30-d Fills per member per month, No.^c^	0.15	0.17	0.14	0.16	0.01 (0.0 to 0.0)^b^	7.1 (2.3 to 11.8)^b^
Higher use, %^c^^,^^d^	5.5	6.9	5.3	6.0	0.7 (0.1 to 1.3)^b^	11.2 (0.6 to 21.8)^b^

Abbreviations: PDC, percentage of days covered; PDL, preventive drug list.

^a^ Annual means and percentages and absolute and relative difference estimates were derived from difference-in-differences generalized estimating equations regression models with a negative binomial or binomial distribution weighted for matching covariates.

^b^ Measured during the middle 8 months of the baseline (months 3-10) and follow-up period (months 15-22) to avoid inaccurate measurement of carryover 90-day fills and changes caused by anticipation of benefit design switches.

^c^ *P* < .05.

^d^ Higher use defined as the percentage of members with diabetes averaging at least 1.0 fill per month over the entire measurement period.

^e^ Residence in census tracts with less than 10% of households below the federal poverty level based on the 2010-2014 American Community Survey.^26^

^f^ Residence in census tracts with 10% or more of households below the federal poverty level based on the 2010-2014 American Community Survey.^26^

Magnitudes of increased antidiabetic agent use were largest among PDL members residing in lower-income areas. For example, 30-day fills of noninsulin agents increased by 10.5% (95% CI, 7.4%-13.6%), and higher use increased by 15.2% (95% CI, 10.6%-19.8%) compared with controls. The corresponding relative increase in insulin use among residents of lower-income areas in the PDL group were 7.1% (95% CI, 2.3%-11.8%) and 11.2% (95% CI, 0.6%-21.8%), respectively.

### Primary Outcome

The PDL transition was associated with an absolute decline of 20.2 (95% CI, −34.3 to −6.2) acute, preventable complication days per 1000 members per year from baseline to follow-up (relative change, −8.4%; 95% CI, −13.9% to −2.8%) compared with controls (Table 4). The PDL members from lower-income areas experienced an absolute decline of 26.1 (95% CI, −45.8 to −6.5) complication days per 1000 members per year (relative change, −10.2%; 95% CI, −17.4% to −3.0%) compared with controls (Figure). We detected no significant changes in complication days among PDL members from higher-income areas (relative change, −6.2%; 95% CI, −14.8% to 2.4%).

**Table 4.** Changes in Acute, Preventable Diabetes Complication Days in the PDL Group vs Control Group From Baseline to Follow-Up^a^

Cohort	Acute, preventable diabetes complication days per 1000 members per year^b^				Change in complication days	
	PDL group		Control group			
	Baseline	Follow-Up	Baseline	Follow-Up	Absolute, per 1000 members per y (95% CI)	Relative, % (95% CI)
Overall	210	221	202	233	−20.2 (−34.3 to −6.2)^c^	−8.4 (−13.9 to −2.8)^c^
Higher income^d^	180	212	172	215	−14.0 (−34.2 to 6.1)	−6.2 (−14.8 to 2.4)
Lower income^e^	236	230	230	249	−26.1 (−45.8 to −6.5)^c^	−10.2 (−17.4 to −3.0)^c^

Abbreviation: PDL, preventive drug list.

^a^ Annual rates and absolute and relative difference estimates were derived from difference-in-differences generalized estimating equation regression models with a negative binomial distribution weighted for matching covariates.

^b^ Includes visits to the outpatient, telehealth, emergency department, observation, and inpatient settings for conditions such as bacterial infections, hyperglycemia-related conditions, and acute vascular events.

^c^ *P* < .05.

^d^ Residence in census tracts with less than 10% of households below the federal poverty level based on the 2010-2014 American Community Survey.^26^

^e^ Residence in census tracts with 10% or more of households below the federal poverty level based on the 2010-2014 American Community Survey.^26^

**Figure.**
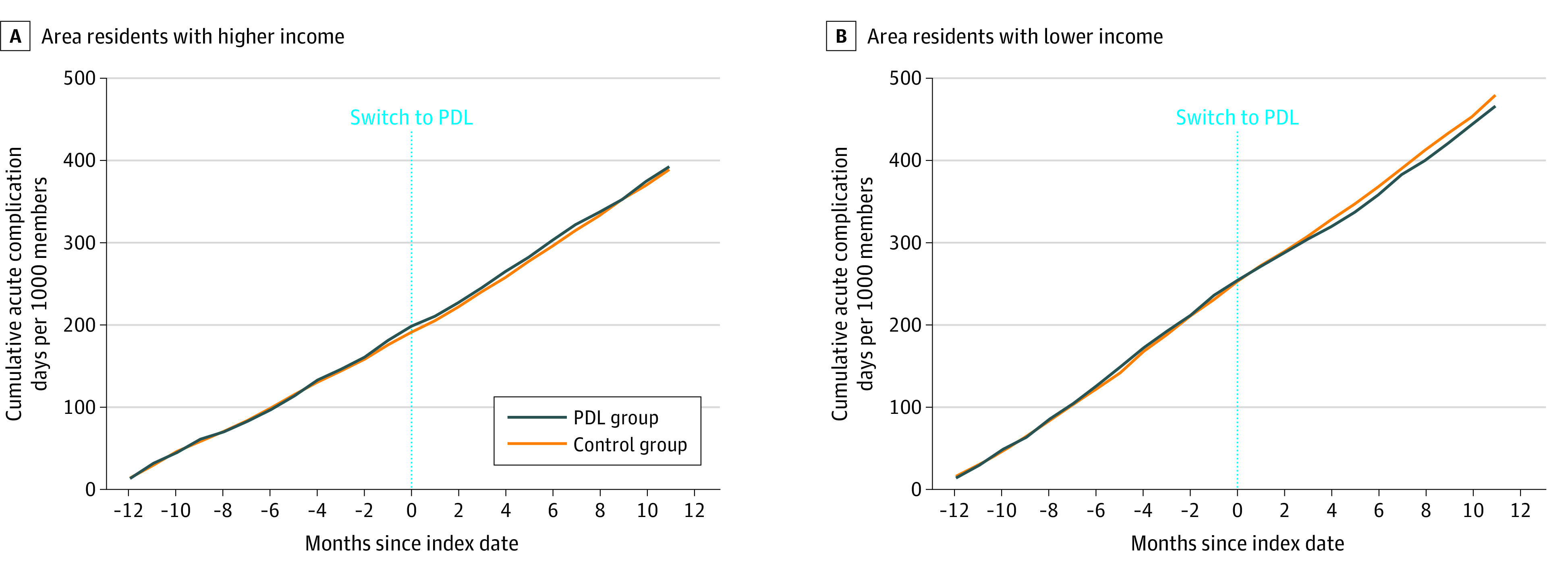
Weighted Cumulative Acute, Preventable Diabetes Complication Days From Baseline to Follow-Up Includes visits to outpatient, telehealth, emergency department, observation, and inpatient settings for conditions such as bacterial infections, hyperglycemia-related conditions, and acute vascular events. Plots display cumulative rates that have been adjusted for weights derived from the match. A, Residence in census tracts with less than 10% of households below the federal poverty level based on the 2010-2014 American Community Survey.^26^ B, Residence in census tracts with 10% or more of households below the federal poverty level based on the 2010-2014 American Community Survey.^26^ PDL indicates preventive drug list.

## Discussion

In this study, patients with diabetes who were enrolled in a value-based medication benefit increased their use of antidiabetic medications and experienced 8.4% fewer acute, preventable diabetes complication days than controls. Preventive drug list members from lower-income areas, who experienced the largest magnitude increases in antidiabetic medication use, experienced a 10.2% decline in complication days. Our results suggest that value-based medication plans might benefit commercially insured patients with diabetes and lower income.

To our knowledge, previous studies have not examined effects of reduced medication cost sharing on diabetes complications, other substantive diabetes-related clinical end points, or patients with low income. In addition, few studies among other populations have assessed health outcomes. The RAND Health Insurance Experiment of the 1970s to 1980s detected improved blood pressure control among participants assigned to lower vs higher cost-sharing levels but did not measure downstream health outcomes.^38^ In a randomized clinical trial of a population of patients who had experienced myocardial infarction, Choudhry et al^17^ found that a cost of $0 for cardioprotective drugs did not improve the primary outcome (first major vascular event or revascularization), although total major vascular events and revascularization declined. Two randomized clinical trials from Canada found small to moderate increases in medication use when drug co-payments were eliminated,^18,39^ with 1 detecting a nonsignificant reduction in incidence of a composite cardiovascular outcome.^18^ The second study found no change in hemoglobin A_1c_ levels,^39^ although it included only approximately 90 treated patients with diabetes per arm and did not examine clinical end points.

Our study therefore adds the key finding that value-based medication cost-sharing reductions may be associated with modest improvement in a relatively common, short-term health outcome among select patients with chronic illness. We hypothesized that PDL members with lower income would experience greater reductions in acute, preventable complication days than counterparts with higher income. Although we found that residents of lower-income areas with PDLs had a larger magnitude of reduction than counterparts residing in higher-income areas, effect estimates did not differ statistically. Nevertheless, our results suggest that targeting out-of-pocket cost reductions to specific populations, in this case patients with diabetes from lower-income areas, might enhance health outcomes. Preventive drug lists might also benefit patients with other chronic illnesses at risk for treatment adherence–related complications, but further research is needed.

### Limitations

Our analyses have several limitations. The findings generalize only to patients with diabetes in commercial health plans. The assignment to PDL coverage was not random but rather was chosen by certain employers. We matched on employer characteristics that could predict this choice, and we minimized potential patient self-selection by limiting to members who were less likely to be able to adopt or not adopt a PDL. Our matching and postmatch weighting produced samples that were nearly identical on measured baseline characteristics. We had no direct measure of PDL exposure and used a claims-based algorithm to impute the presence of a PDL, potentially introducing error in study group assignment. However, during a given employer’s benefit year, we were able to detect when PDL-targeted medications had $0 out-of-pocket costs and non-PDL medications had typical cost-sharing levels, generating a contrast with face validity for flagging PDL adoption. In addition, the medication expenditure patterns that we observed before and after the switch (Table 2) suggest that our algorithms reliably identified employers that switched to PDLs. We required that members in our study have continuous enrollment for 1 year before and after the index date, which could reduce generalizability and bias results toward the null if PDL enrollment reduced mortality and disenrollment. Finally, this study only examined outcomes associated with PDL enrollment over a single year; longer-term studies that include additional clinical outcomes will require larger samples.

## Conclusions

In this cohort study, a value-based medication benefit that lowers antidiabetic medication out-of-pocket costs was associated with small to moderate increases in antidiabetic medication use and an 8.4% reduction in acute, preventable diabetes complication days. Patients with diabetes residing in lower-income areas experienced a 10.2% relative reduction in complication days after enrollment in PDL plans. Our results may support a strategy of incentivizing adoption of targeted cost-sharing reductions among commercially insured patients with diabetes and lower income to enhance health outcomes.^40^

## Notes

Supplement 1.
eMethods
**eTable 1.** Number of Medications Listed on the 2017 Core PDL and Expanded PDL by Therapeutic Category **eTable 2.** Medications on the 2017 Expanded PDL by Therapeutic Category **eTable 3.** Steps in Imputing Presence of PDL at the Account Level and Determining Full Replacement of No PDL With PDL at Linked Account Level **eTable 4.** Diagnosis and Medication Codes Used to Define Patients With Diabetes **eFigure.** Decision Algorithm for Classifying the Primary *ICD* Diagnosis at Outpatient and High-Acuity Visits as Complications of Diabetes Caused by Short-Term Reduced Care Access, Emphasizing Specificity **eTable 5.** Ranked Frequency of Categories of Acute, Preventable Complication Days at Baseline **eTable 6.** Tests of Baseline Trends in Acute, Preventable Complication Days Between PDL and Control Groups
eReferences

Supplement 2.
Data Sharing Statement
