# Supplementary material for: Acute Diabetes Complications After Transition to a Value-Based Medication Benefit
Source: JAMA Health Forum. 2024 Feb 9;5(2):e235309. doi: 10.1001/jamahealthforum.2023.5309 (PMC10858396; doi:10.1001/jamahealthforum.2023.5309)
Supplement: Supplement 1. — eMethods eTable 1. Number of Medications Listed on the 2017 Core PDL and Expanded PDL by Therapeutic Category eTable 2. Medications on the 2017 Expanded PDL by Therapeutic Category eTable 3. Steps in Imputing Presence of PDL at the Account Level and Determining Full Replacement of No PDL With PDL at Linked Account Level eTable 4. Diagnosis and Medication Codes Used to Define Patients With Diabetes eFigure. Decision Algorithm for Classifying the Primary ICD Diagnosis at Outpatient and High-Acuity Visits as Complications of Diabetes Caused by Short-Term Reduced Care Access, Emphasizing Specificity eTable 5. Ranked Frequency of Categories of Acute, Preventable Complication Days at Baseline eTable 6. Tests of Baseline Trends in Acute, Preventable Complication Days Between PDL and Control Groups eReferences [file jamahealthforum-e235309-s001.pdf]

Supplemental Online Content

Wharam JF, Argetsinger S, Lakoma M, Zhang F, Ross-Denan D. Acute diabetes complications after transition to a value-based medication benefit. *JAMA Health Forum*. 2024;5(2):e235309.
doi:10.1001/jamahealthforum.2023.5309

eMethods

eTable 1. Number of Medications Listed on the 2017 Core PDL and Expanded PDL by Therapeutic Category

eTable 2. Medications on the 2017 Expanded PDL by Therapeutic Category

eTable 3. Steps in Imputing Presence of PDL at the Account Level and Determining Full Replacement of No PDL With PDL at Linked Account Level

eTable 4. Diagnosis and Medication Codes Used to Define Patients With Diabetes

eFigure. Decision Algorithm for Classifying the Primary *ICD* Diagnosis at Outpatient and High-Acuity Visits as Complications of Diabetes Caused by Short-Term Reduced Care Access, Emphasizing Specificity

eTable 5. Ranked Frequency of Categories of Acute, Preventable Complication Days at Baseline

eTable 6. Tests of Baseline Trends in Acute, Preventable Complication Days Between PDL and Control Groups

eReferences

This supplementary material has been provided by the authors to give readers additional information about their work.

© 2024 Wharam JF et al. *JAMA Health Forum.*

I. eMethods

A. Contents of the 2017 Core and Expanded Preventive Drug Lists

Employers adopt Preventive Drug Lists (PDL) to reduce member cost sharing for key therapeutic classes of preventive medications. These PDLs come in several varieties, but the two most common are a standard Core PDL and an optional Expanded PDL, which includes all medicines on the Core List plus several other key therapeutic categories, including insulin and non-insulin antidiabetic agents. The specific products included on these PDLs are modified annually. eTable 1 summarizes the numbers of products listed by brand name or by generic name in the 2017 Core and Expanded Lists. eTable 2 displays medications on the 2017 Expanded PDL by therapeutic category.

eTable 1. Number of Medications Listed on the 2017 Core PDL and Expanded PDL by Therapeutic Category

	Core List (N=316)		Expanded List (n=560)	
**Therapeutic Category**	**Generic**	**Brand**	**Generic**	**Brand**
CVD - Blood Pressure	89	108	89	108
CVD - Lipid Lowering	19	29	19	29
CVD - Clotting/Platelet Therapy	8	16	8	16
Osteoporosis, Menopause	6	11	6	11
Immunosuppression	6	12	6	12
Breast Cancer Prevention	4	5	4	5
Prenatal & Pediatric	2	1	2	1
Diabetes - Insulin				19
Diabetes - Non-insulin			18	46
Diabetes - Supplies			2	2
Respiratory - Asthma/COPD			14	51
CNS - Psychosis, Depression			16	15
CNS - Multiple Sclerosis				11
Anti-Infectives - HIV/AIDS			9	41
**Total**	**134**	**182**	**193**	**367**

eTable 2. Medications on the 2017 Expanded PDL by Therapeutic Category

Drug Listed[1]	Generic Equivalent	Therapeutic Category	First Listed	On Core List	Excludable[2]
abacavir	abacavir sulfate	Anti-Infectives - HIV/AIDS	2014		0
abacavir-lamivudine-zidovudine	abacavir/lamivudine/zidovudine	Anti-Infectives - HIV/AIDS	2014		0
Abilify	Aripiprazole	CNS - Psychosis	2020		1
acarbose	Acarbose	Diabetes - Non-insulin	2009		0
Accolate	Zafirlukast	Respiratory - Asthma/COPD	2008		0
Accuneb	albuterol sulfate	Respiratory - Asthma/COPD	2008		0
Accupril	quinapril hcl	CVD - Blood Pressure	2008	1	0
Accuretic	quinapril/hydrochlorothiazide	CVD - Blood Pressure	2008	1	0
acebutolol	acebutolol hcl	CVD - Blood Pressure	2008	1	0
Aceon	perindopril erbumine	CVD - Blood Pressure	2008	1	0
Actonel	risedronate sodium	Osteoporosis, Menopause	2008	1	0
ACTOplus Met	pioglitazone hcl/metformin hcl	Diabetes - Non-insulin	2008		0
ACTOplus Met XR	pioglitazone hcl/metformin hcl	Diabetes - Non-insulin	2012		0
Actos	pioglitazone hcl	Diabetes - Non-insulin	2008		1
Adalat CC	nifedipine	CVD - Blood Pressure	2008	1	0

© 2024 Wharam JF et al. *JAMA Health Forum.*

eTable 2. Medications on the 2017 Expanded PDL by Therapeutic Category

Drug Listed[1]	Generic Equivalent	Therapeutic Category	First Listed	On Core List	Excludable[2]
Advair Diskus	fluticasone propion/salmeterol	Respiratory - Asthma/COPD	2008		0
Advair HFA	fluticasone propion/salmeterol	Respiratory - Asthma/COPD	2010		0
Aerospan	flunisolide	Respiratory - Asthma/COPD	2015		0
Afeditab	nifedipine	CVD - Blood Pressure	2009	1	0
Afrezza	insulin regular, human	Diabetes - Insulin	2015		1
Aggrenox	aspirin/dipyridamole	CVD - Clotting/Platelet Therapy	2008	1	0
albuterol nebulized solution	albuterol sulfate	Respiratory - Asthma/COPD	2008		0
albuterol oral tablet	albuterol sulfate	Respiratory - Asthma/COPD	2011		0
Aldactazide	spironolact/hydrochlorothiazid	CVD - Blood Pressure	2011	1	0
Aldactone	spironolactone	CVD - Blood Pressure	2008	1	0
alendronate	alendronate sodium	Osteoporosis, Menopause	2008	1	0
Altace	ramipril	CVD - Blood Pressure	2008	1	0
Altoprev	lovastatin	CVD - Lipid Lowering	2008	1	1
Alvesco	ciclesonide	Respiratory - Asthma/COPD	2010		0
Amaryl	glimepiride	Diabetes - Non-insulin	2008		0
amiloride	amiloride hcl	CVD - Blood Pressure	2011	1	0
amiloride-hydrochlorothiazide	amiloride/hydrochlorothiazide	CVD - Blood Pressure	2008	1	0
aminophylline	aminophylline	Respiratory - Asthma/COPD	2011		0
amlodipine	amlodipine besylate	CVD - Blood Pressure	2008	1	0
amlodipine-benazepril	amlodipine besylate/benazepril	CVD - Blood Pressure	2008	1	0
amlodipine-valsartan	amlodipine besylate/valsartan	CVD - Blood Pressure	2015	1	1
amlodipine-valsartan-hydrochlorothiazide	amlodipine/valsartan/hcthiazid	CVD - Blood Pressure	2015	1	1
Amturnide	aliskiren/amlodipin/hcthiazide	CVD - Blood Pressure	2012	1	1
anastrozole	anastrozole	Breast Cancer Prevention	2011	1	0
Anoro Ellipta	umeclidinium brm/vilanterol tr	Respiratory - Asthma/COPD	2014		0
Antara	fenofibrate,micronized	CVD - Lipid Lowering	2008	1	1
Apidra	insulin glulisine	Diabetes - Insulin	2008		0
Aptivus	tipranavir	Anti-Infectives - HIV/AIDS	2011		0
Arcapta Neohaler	indacaterol maleate	Respiratory - Asthma/COPD	2013		0
Arimidex	anastrozole	Breast Cancer Prevention	2011	1	1
aripiprazole	aripiprazole	CNS - Psychosis	2016		0
Arixtra	fondaparinux sodium	CVD - Clotting/Platelet Therapy	2011	1	0
Arnuity Ellipta	fluticasone furoate	Respiratory - Asthma/COPD	2015		1
Aromasin	exemestane	Breast Cancer Prevention	2011	1	0
Asmanex HFA	mometasone furoate	Respiratory - Asthma/COPD	2015		0
Asmanex Twisthaler	mometasone furoate	Respiratory - Asthma/COPD	2008		0
Astagraf XL	tacrolimus	Immunosuppression	2014	1	1
Atacand	candesartan cilexetil	CVD - Blood Pressure	2008	1	0
Atacand HCT	candesartan/hydrochlorothiazid	CVD - Blood Pressure	2008	1	0
Atelvia	risedronate sodium	Osteoporosis, Menopause	2013	1	1
atenolol	atenolol	CVD - Blood Pressure	2008	1	0
atenolol-chlorthalidone	atenolol/chlorthalidone	CVD - Blood Pressure	2008	1	0
atorvastatin	atorvastatin calcium	CVD - Lipid Lowering	2013	1	0
Atripla	efavirenz/emtricit/tenofovr df	Anti-Infectives - HIV/AIDS	2011		0
Atrovent HFA	ipratropium bromide	Respiratory - Asthma/COPD	2008		0
Aubagio	teriflunomide	CNS - Multiple Sclerosis	2014		0
Avalide	irbesartan/hydrochlorothiazide	CVD - Blood Pressure	2008	1	0
Avandia	rosiglitazone maleate	Diabetes - Non-insulin	2008		0

© 2024 Wharam JF et al. *JAMA Health Forum.*

eTable 2. Medications on the 2017 Expanded PDL by Therapeutic Category

Drug Listed[1]	Generic Equivalent	Therapeutic Category	First Listed	On Core List	Excludable[2]
Avapro	irbesartan	CVD - Blood Pressure	2008	1	0
Avonex	interferon beta-1a	CNS - Multiple Sclerosis	2011		0
Azasan	azathioprine	Immunosuppression	2011	1	0
azathioprine	azathioprine	Immunosuppression	2011	1	0
Azor	amlodipine bes/olmesartan med	CVD - Blood Pressure	2008	1	1
Basaglar	insulin glargine,hum.rec.anlog	Diabetes - Insulin	2017		0
benazepril	benazepril hcl	CVD - Blood Pressure	2008	1	0
benazepril-hydrochlorothiazide	benazepril/hydrochlorothiazide	CVD - Blood Pressure	2008	1	0
Benicar	olmesartan medoxomil	CVD - Blood Pressure	2008	1	0
Benicar HCT	olmesartan/hydrochlorothiazide	CVD - Blood Pressure	2008	1	0
Betaseron	interferon beta-1b	CNS - Multiple Sclerosis	2011		0
betaxolol**	betaxolol hcl	CVD - Blood Pressure	2011	1	0
Bevespi Aerosphere	glycopyrrolate/formoterol fum	Respiratory - Asthma/COPD	2017		0
Bidil	isosorbide dinit/hydralazine	CVD - Blood Pressure	2008	1	0
Binosto	alendronate sodium	Osteoporosis, Menopause	2014	1	1
bisoprolol	bisoprolol fumarate	CVD - Blood Pressure	2008	1	0
bisoprolol-hydrochlorothiazide	bisoprolol/hydrochlorothiazide	CVD - Blood Pressure	2008	1	0
Boniva	ibandronate sodium	Osteoporosis, Menopause	2008	1	0
Breo Ellipta	fluticasone/vilanterol	Respiratory - Asthma/COPD	2014		0
Brilinta	ticagrelor	CVD - Clotting/Platelet Therapy	2012	1	0
Brovana	arformoterol tartrate	Respiratory - Asthma/COPD	2008		0
budesonide	budesonide	Respiratory - Asthma/COPD	2008		0
bumetanide	bumetanide	CVD - Blood Pressure	2008	1	0
Bydureon	exenatide microspheres	Diabetes - Non-insulin	2013		0
Byetta	exenatide	Diabetes - Non-insulin	2008		0
Bystolic	nebivolol hcl	CVD - Blood Pressure	2008	1	0
Byvalson	nebivolol hcl/valsartan	CVD - Blood Pressure	2017	1	1
Calan	verapamil hcl	CVD - Blood Pressure	2008	1	0
Calan SR	verapamil hcl	CVD - Blood Pressure	2008	1	0
calcitonin (salmon)	calcitonin,salmon,synthetic	Osteoporosis, Menopause	2010	1	0
candesartan	candesartan cilexetil	CVD - Blood Pressure	2014	1	0
candesartan-hydrochlorothiazide	candesartan/hydrochlorothiazid	CVD - Blood Pressure	2014	1	0
captopril	captopril	CVD - Blood Pressure	2008	1	0
captopril-hydrochlorothiazide	captopril/hydrochlorothiazide	CVD - Blood Pressure	2008	1	0
Cardene SR	nicardipine hcl	CVD - Blood Pressure	2008	1	0
Cardizem CD	diltiazem hcl	CVD - Blood Pressure	2008	1	1
Cardizem	diltiazem hcl	CVD - Blood Pressure	2011	1	1
Cardizem LA	diltiazem hcl	CVD - Blood Pressure	2008	1	1
Cardura	doxazosin mesylate	CVD - Blood Pressure	2008	1	0
Cartia XT	diltiazem hcl	CVD - Blood Pressure	2008	1	0
carvedilol	carvedilol	CVD - Blood Pressure	2008	1	0
Catapres	clonidine hcl	CVD - Blood Pressure	2008	1	0
Catapres TTS	clonidine	CVD - Blood Pressure	2008	1	0
Cellcept	mycophenolate mofetil	Immunosuppression	2011	1	0
chlorothiazide	chlorothiazide	CVD - Blood Pressure	2008	1	0
chlorpromazine	chlorpromazine hcl	CNS - Psychosis	2008		0
cholestyramine	cholestyramine	CVD - Lipid Lowering	2008	1	0
cholestyramine light	cholestyramine/aspartame	CVD - Lipid Lowering	2008	1	0
choline fenofibrate	fenofibric acid (choline)	CVD - Lipid Lowering	2014	1	1

© 2024 Wharam JF et al. *JAMA Health Forum.*

eTable 2. Medications on the 2017 Expanded PDL by Therapeutic Category

Drug Listed[1]	Generic Equivalent	Therapeutic Category	First Listed	On Core List	Excludable[2]
cilostazol	cilostazol	CVD - Clotting/Platelet Therapy	2011	1	0
clonidine	clonidine	CVD - Blood Pressure	2008	1	0
clonidine patch	clonidine	CVD - Blood Pressure	2011	1	0
clopidogrel	clopidogrel bisulfate	CVD - Clotting/Platelet Therapy	2008	1	0
Clorpres	clonidine hcl/chlorthalidone	CVD - Blood Pressure	2009	1	0
clozapine	clozapine	CNS - Psychosis	2008		0
Clozaril	clozapine	CNS - Psychosis	2008		0
Colestid	colestipol hcl	CVD - Lipid Lowering	2008	1	0
colestipol	colestipol hcl	CVD - Lipid Lowering	2008	1	0
Combivent Respimat	ipratropium/albuterol sulfate	Respiratory - Asthma/COPD	2014		0
Combivir	lamivudine/zidovudine	Anti-Infectives - HIV/AIDS	2011		0
Complera	emtricita/rilpivirine/tenof df	Anti-Infectives - HIV/AIDS	2012		0
Copaxone	glatiramer acetate	CNS - Multiple Sclerosis	2011		0
Coreg	carvedilol	CVD - Blood Pressure	2008	1	0
Coreg CR	carvedilol phosphate	CVD - Blood Pressure	2008	1	1
Corgard	nadolol	CVD - Blood Pressure	2008	1	0
Corzide	nadolol/bendroflumethiazide	CVD - Blood Pressure	2008	1	0
Coumadin	warfarin sodium	CVD - Clotting/Platelet Therapy	2008	1	0
Covera HS	verapamil hcl	CVD - Blood Pressure	2008	1	0
Cozaar	losartan potassium	CVD - Blood Pressure	2008	1	0
Crestor	rosuvastatin calcium	CVD - Lipid Lowering	2008	1	1
Crixivan	indinavir sulfate	Anti-Infectives - HIV/AIDS	2011		0
cromolyn	cromolyn sodium	Respiratory - Asthma/COPD	2008		0
Cycloset	bromocriptine mesylate	Diabetes - Non-insulin	2013		0
cyclosporine	cyclosporine	Immunosuppression	2011	1	0
Daliresp	roflumilast	Respiratory - Asthma/COPD	2013		0
Demadex	torsemide	CVD - Blood Pressure	2008	1	0
Descovy	emtricitabine/tenofov alafenam	Anti-Infectives - HIV/AIDS	2017		0
Diabeta	glyburide	Diabetes - Non-insulin	2008		0
diabetic testing - lancets	diabetic testing - lancets	Diabetes - Supplies	2011		0
didanosine	didanosine	Anti-Infectives - HIV/AIDS	2011		0
Didronel	etidronate disodium	Osteoporosis, Menopause	2010	1	0
Dilacor XR	diltiazem hcl	CVD - Blood Pressure	2008	1	0
Dilt CD	diltiazem hcl	CVD - Blood Pressure	2011	1	0
Dilt XR	diltiazem hcl	CVD - Blood Pressure	2011	1	0
Diltia XT	diltiazem hcl	CVD - Blood Pressure	2008	1	0
diltiazem	diltiazem hcl	CVD - Blood Pressure	2008	1	0
diltiazem ER	diltiazem hcl	CVD - Blood Pressure	2008	1	0
Diltzac ER	diltiazem hcl	CVD - Blood Pressure	2011	1	0
Diovan	valsartan	CVD - Blood Pressure	2008	1	1
Diovan HCT	valsartan/hydrochlorothiazide	CVD - Blood Pressure	2008	1	1
dipyridamole	dipyridamole	CVD - Clotting/Platelet Therapy	2010	1	0
Diuril	chlorothiazide	CVD - Blood Pressure	2011	1	0
doxazosin	doxazosin mesylate	CVD - Blood Pressure	2008	1	0
Duetact	pioglitazone hcl/glimepiride	Diabetes - Non-insulin	2010		0
Dulera	mometasone/formoterol	Respiratory - Asthma/COPD	2011		0
Duoneb	ipratropium/albuterol sulfate	Respiratory - Asthma/COPD	2008		0
Dutoprol	metoprolol su/hydrochlorothiaz	CVD - Blood Pressure	2008	1	0

© 2024 Wharam JF et al. *JAMA Health Forum.*

eTable 2. Medications on the 2017 Expanded PDL by Therapeutic Category

Drug Listed[1]	Generic Equivalent	Therapeutic Category	First Listed	On Core List	Excludable[2]
Dyazide	triamterene/hydrochlorothiazid	CVD - Blood Pressure	2008	1	0
Dynacirc CR	isradipine	CVD - Blood Pressure	2008	1	0
Dyrenium	triamterene	CVD - Blood Pressure	2011	1	0
Edarbi	azilsartan medoxomil	CVD - Blood Pressure	2013	1	0
Edarbyclor	azilsartan med/chlorthalidone	CVD - Blood Pressure	2013	1	0
Edecrin	ethacrynic acid	CVD - Blood Pressure	2009	1	0
Edurant	rilpivirine hcl	Anti-Infectives - HIV/AIDS	2013		0
Effient	prasugrel hcl	CVD - Clotting/Platelet Therapy	2010	1	0
Eliquis	apixaban	CVD - Clotting/Platelet Therapy	2014	1	0
Elixophyllin	theophylline anhydrous	Respiratory - Asthma/COPD	2008		0
Emtriva	emtricitabine	Anti-Infectives - HIV/AIDS	2011		0
enalapril	enalapril maleate	CVD - Blood Pressure	2008	1	0
enalapril-hydrochlorothiazide	enalapril/hydrochlorothiazide	CVD - Blood Pressure	2008	1	0
enoxaparin	enoxaparin sodium	CVD - Clotting/Platelet Therapy	2011	1	0
Envarsus XR	tacrolimus	Immunosuppression	2017	1	1
Epaned	enalapril maleate	CVD - Blood Pressure	2014	1	1
Epivir	lamivudine	Anti-Infectives - HIV/AIDS	2011		0
eplerenone	eplerenone	CVD - Blood Pressure	2011	1	0
eprosartan	eprosartan mesylate	CVD - Blood Pressure	2013	1	0
Epzicom	abacavir sulfate/lamivudine	Anti-Infectives - HIV/AIDS	2011		0
ethacrynic acid	ethacrynic acid	CVD - Blood Pressure	2008	1	0
etidronate	etidronate disodium	Osteoporosis, Menopause	2010	1	0
Evista	raloxifene hcl	Osteoporosis, Menopause	2008	1	1
Evotaz	atazanavir sulfate/cobicistat	Anti-Infectives - HIV/AIDS	2015		1
exemestane	exemestane	Breast Cancer Prevention	2012	1	0
Exforge	amlodipine besylate/valsartan	CVD - Blood Pressure	2008	1	1
Exforge HCT	amlodipine/valsartan/hcthiazid	CVD - Blood Pressure	2010	1	1
Extavia	interferon beta-1b	CNS - Multiple Sclerosis	2011		1
Fanapt	iloperidone	CNS - Psychosis	2010		0
Fareston	toremifene citrate	Breast Cancer Prevention	2011	1	0
Farxiga	dapagliflozin propanediol	Diabetes - Non-insulin	2014		0
FazaClo	clozapine	CNS - Psychosis	2008		0
felodipine ER	felodipine	CVD - Blood Pressure	2008	1	0
Femara	letrozole	Breast Cancer Prevention	2008	1	1
fenofibrate 40,48,120,145 mg tablet	fenofibrate	CVD - Lipid Lowering	2014	1	1
fenofibrate 43,50,67,130,134,150,200 mg capsule	fenofibrate	CVD - Lipid Lowering	2015	1	1
fenofibrate 54,160 mg tablet	fenofibrate	CVD - Lipid Lowering	2015	1	0
fenofibric acid	fenofibric acid (choline)	CVD - Lipid Lowering	2014	1	1
Fenoglide	fenofibrate	CVD - Lipid Lowering	2010	1	1
Fibricor	fenofibric acid	CVD - Lipid Lowering	2010	1	1
Flovent Diskus	fluticasone propionate	Respiratory - Asthma/COPD	2009		0
Flovent HFA	fluticasone propionate	Respiratory - Asthma/COPD	2008		0
fluphenazine	fluphenazine hcl	CNS - Psychosis	2008		0
fluvastatin	fluvastatin sodium	CVD - Lipid Lowering	2013	1	0
fluvastatin ER	fluvastatin sodium	CVD - Lipid Lowering	2017	1	0
fondaparinux	fondaparinux sodium	CVD - Clotting/Platelet Therapy	2012	1	0

© 2024 Wharam JF et al. *JAMA Health Forum.*

eTable 2. Medications on the 2017 Expanded PDL by Therapeutic Category

Drug Listed[1]	Generic Equivalent	Therapeutic Category	First Listed	On Core List	Excludable[2]
Foradil	formoterol fumarate	Respiratory - Asthma/COPD	2008		0
Fortamet	metformin hcl	Diabetes - Non-insulin	2009		1
Forteo	teriparatide	Osteoporosis, Menopause	2008	1	0
Fortical	calcitonin,salmon,synthetic	Osteoporosis, Menopause	2008	1	0
Fosamax	alendronate sodium	Osteoporosis, Menopause	2008	1	0
Fosamax Plus D	alendronate sodium	Osteoporosis, Menopause	2008	1	0
fosinopril	fosinopril sodium	CVD - Blood Pressure	2008	1	0
fosinopril-hydrochlorothiazide	fosinopril/hydrochlorothiazide	CVD - Blood Pressure	2008	1	0
Fragmin	dalteparin sodium,porcine	CVD - Clotting/Platelet Therapy	2011	1	0
furosemide	furosemide	CVD - Blood Pressure	2008	1	0
Fuzeon	enfuvirtide	Anti-Infectives - HIV/AIDS	2011		0
Gastrocrom	cromolyn sodium	Respiratory - Asthma/COPD	2011		0
gemfibrozil	gemfibrozil	CVD - Lipid Lowering	2008	1	0
Gengraf	cyclosporine, modified	Immunosuppression	2011	1	0
Genvoya	elviteg/cob/emtri/tenof alafen	Anti-Infectives - HIV/AIDS	2017		0
Geodon	ziprasidone hcl	CNS - Psychosis	2008		1
Gilenya	fingolimod hcl	CNS - Multiple Sclerosis	2012		0
Glatopa	glatiramer acetate	CNS - Multiple Sclerosis	2016		1
glimepiride	glimepiride	Diabetes - Non-insulin	2008		0
glipizide	glipizide	Diabetes - Non-insulin	2008		0
glipizide ER	glipizide	Diabetes - Non-insulin	2011		0
glipizide-metformin	glipizide/metformin hcl	Diabetes - Non-insulin	2008		0
Glucophage	metformin hcl	Diabetes - Non-insulin	2008		0
Glucophage XR	metformin hcl	Diabetes - Non-insulin	2008		0
Glucotrol	glipizide	Diabetes - Non-insulin	2008		0
Glucotrol XL	glipizide	Diabetes - Non-insulin	2008		0
Glucovance	glyburide/metformin hcl	Diabetes - Non-insulin	2008		0
Glumetza	metformin hcl	Diabetes - Non-insulin	2008		1
glyburide	glyburide	Diabetes - Non-insulin	2008		0
glyburide micronized	glyburide,micronized	Diabetes - Non-insulin	2011		0
glyburide-metformin	glyburide/metformin hcl	Diabetes - Non-insulin	2008		0
Glynase	glyburide,micronized	Diabetes - Non-insulin	2008		0
Glyset	miglitol	Diabetes - Non-insulin	2008		0
Glyxambi	empagliflozin/linagliptin	Diabetes - Non-insulin	2015		1
guanfacine	guanfacine hcl	CVD - Blood Pressure	2008	1	0
haloperidol	haloperidol	CNS - Psychosis	2008		0
heparin	heparin	CVD - Clotting/Platelet Therapy	2011	1	0
Humalog	insulin lispro	Diabetes - Insulin	2008		0
Humalog Mix 50/50	insulin lispro protamin/lispro	Diabetes - Insulin	2009		0
Humalog Mix 75/25	insulin lispro protamin/lispro	Diabetes - Insulin	2008		0
Humulin 50/50	insulin nph hum/reg insulin hm	Diabetes - Insulin	2008		0
Humulin 70/30	insulin nph hum/reg insulin hm	Diabetes - Insulin	2008		0
Humulin N	insulin nph human isophane	Diabetes - Insulin	2008		0
Humulin R	insulin regular, human	Diabetes - Insulin	2008		0
hydralazine	hydralazine hcl	CVD - Blood Pressure	2008	1	0
hydrochlorothiazide	hydrochlorothiazide	CVD - Blood Pressure	2008	1	0
Hyzaar	losartan/hydrochlorothiazide	CVD - Blood Pressure	2008	1	0
ibandronate	ibandronate sodium	Osteoporosis, Menopause	2013	1	0
Imuran	azathioprine	Immunosuppression	2011	1	0
Incruse Ellipta	umeclidinium bromide	Respiratory - Asthma/COPD	2015		0

© 2024 Wharam JF et al. *JAMA Health Forum.*

eTable 2. Medications on the 2017 Expanded PDL by Therapeutic Category

Drug Listed[1]	Generic Equivalent	Therapeutic Category	First Listed	On Core List	Excludable[2]
indapamide	indapamide	CVD - Blood Pressure	2008	1	0
Inderal	propranolol hcl	CVD - Blood Pressure	2008	1	0
Inderal LA	propranolol hcl	CVD - Blood Pressure	2008	1	0
Innopran XL	propranolol hcl	CVD - Blood Pressure	2008	1	0
Inspra	eplerenone	CVD - Blood Pressure	2011	1	0
insulin needles/syringes	insulin needles/syringes	Diabetes - Supplies	2011		0
Intelence	etravirine	Anti-Infectives - HIV/AIDS	2011		0
Invega	paliperidone	CNS - Psychosis	2008		1
Invirase	saquinavir mesylate	Anti-Infectives - HIV/AIDS	2011		0
Invokamet	canagliflozin/metformin hcl	Diabetes - Non-insulin	2015		0
Invokana	canagliflozin	Diabetes - Non-insulin	2014		0
ipratropium	ipratropium bromide	Respiratory - Asthma/COPD	2008		0
ipratropium/albuterol	ipratropium/albuterol sulfate	Respiratory - Asthma/COPD	2008		0
irbesartan	irbesartan	CVD - Blood Pressure	2013	1	0
irbesartan-hydrochlorothiazide	irbesartan/hydrochlorothiazide	CVD - Blood Pressure	2013	1	0
Isentress	raltegravir potassium	Anti-Infectives - HIV/AIDS	2011		0
Isoptin SR	verapamil hcl	CVD - Blood Pressure	2008	1	0
isradipine	isradipine	CVD - Blood Pressure	2008	1	0
Jantoven	warfarin sodium	CVD - Clotting/Platelet Therapy	2010	1	0
Janumet	sitagliptin phos/metformin hcl	Diabetes - Non-insulin	2008		0
Janumet XR	sitagliptin phos/metformin hcl	Diabetes - Non-insulin	2013		0
Januvia	sitagliptin phosphate	Diabetes - Non-insulin	2008		0
Jardiance	empagliflozin	Diabetes - Non-insulin	2015		0
Jentadueto	linagliptin/metformin hcl	Diabetes - Non-insulin	2013		0
Jentadueto XR	linagliptin/metformin hcl	Diabetes - Non-insulin	2017		1
Kaletra	lopinavir/ritonavir	Anti-Infectives - HIV/AIDS	2011		0
Kazano	alogliptin benz/metformin hcl	Diabetes - Non-insulin	2014		0
Kombiglyze XR	saxagliptin hcl/metformin hcl	Diabetes - Non-insulin	2012		0
labetalol	labetalol hcl	CVD - Blood Pressure	2008	1	0
lamivudine	lamivudine	Anti-Infectives - HIV/AIDS	2014		0
lamivudine-zidovudine	lamivudine/zidovudine	Anti-Infectives - HIV/AIDS	2014		0
Lantus	insulin glargine,hum.rec.anlog	Diabetes - Insulin	2008		1
Lasix	furosemide	CVD - Blood Pressure	2008	1	0
Latuda	lurasidone hcl	CNS - Psychosis	2009		0
Lescol	fluvastatin sodium	CVD - Lipid Lowering	2008	1	0
Lescol XL	fluvastatin sodium	CVD - Lipid Lowering	2008	1	1
letrozole	letrozole	Breast Cancer Prevention	2012	1	0
levalbuterol nebulized solution	levalbuterol hcl	Respiratory - Asthma/COPD	2010		1
Levatol	penbutolol sulfate	CVD - Blood Pressure	2008	1	0
Levemir	insulin detemir	Diabetes - Insulin	2008		0
Lexiva	fosamprenavir calcium	Anti-Infectives - HIV/AIDS	2011		0
Lipitor	atorvastatin calcium	CVD - Lipid Lowering	2008	1	1
Lipofen	fenofibrate	CVD - Lipid Lowering	2009	1	1
lisinopril	lisinopril	CVD - Blood Pressure	2008	1	0
lisinopril-hydrochlorothiazide	lisinopril/hydrochlorothiazide	CVD - Blood Pressure	2008	1	0
Livalo	pitavastatin calcium	CVD - Lipid Lowering	2010	1	0
Lofibra	fenofibrate	CVD - Lipid Lowering	2008	1	1
Lopid	gemfibrozil	CVD - Lipid Lowering	2008	1	0
Lopressor	metoprolol tartrate	CVD - Blood Pressure	2008	1	0
Lopressor HCT	metoprolol/hydrochlorothiazide	CVD - Blood Pressure	2008	1	0

© 2024 Wharam JF et al. *JAMA Health Forum.*

eTable 2. Medications on the 2017 Expanded PDL by Therapeutic Category

Drug Listed[1]	Generic Equivalent	Therapeutic Category	First Listed	On Core List	Excludable[2]
losartan	losartan potassium	CVD - Blood Pressure	2011	1	0
losartan hydrochlorothiazide	losartan/hydrochlorothiazide	CVD - Blood Pressure	2011	1	0
Lotensin	benazepril hcl	CVD - Blood Pressure	2008	1	0
Lotensin HCT	benazepril/hydrochlorothiazide	CVD - Blood Pressure	2008	1	0
Lotrel	amlodipine besylate/benazepril	CVD - Blood Pressure	2008	1	0
lovastatin	lovastatin	CVD - Lipid Lowering	2008	1	0
Lovaza	omega-3 acid ethyl esters	CVD - Lipid Lowering	2009	1	1
Lovenox	enoxaparin sodium	CVD - Clotting/Platelet Therapy	2011	1	0
loxapine	loxapine succinate	CNS - Psychosis	2008		0
Lufyllin	dyphylline	Respiratory - Asthma/COPD	2008		0
Matzim LA	diltiazem hcl	CVD - Blood Pressure	2014	1	0
Mavik	trandolapril	CVD - Blood Pressure	2008	1	0
Maxzide	triamterene/hydrochlorothiazid	CVD - Blood Pressure	2008	1	0
metaproterenol	metaproterenol sulfate	Respiratory - Asthma/COPD	2008		0
metformin	metformin hcl	Diabetes - Non-insulin	2008		0
metformin ER	metformin hcl	Diabetes - Non-insulin	2011		1
methyclothiazide	methyclothiazide	CVD - Blood Pressure	2008	1	0
methyldopa	methyldopa	CVD - Blood Pressure	2008	1	0
methyldopa-hydrochlorothiazide	methyldopa/hydrochlorothiazide	CVD - Blood Pressure	2008	1	0
metolazone	metolazone	CVD - Blood Pressure	2008	1	0
metoprolol succinate	metoprolol succinate	CVD - Blood Pressure	2008	1	0
metoprolol tartrate	metoprolol tartrate	CVD - Blood Pressure	2008	1	0
metoprolol-hydrochlorothiazide	metoprolol su/hydrochlorothiaz	CVD - Blood Pressure	2008	1	0
Mevacor	lovastatin	CVD - Lipid Lowering	2008	1	0
Miacalcin	calcitonin,salmon,synthetic	Osteoporosis, Menopause	2008	1	0
Micardis	telmisartan	CVD - Blood Pressure	2008	1	1
Micardis HCT	telmisartan/hydrochlorothiazid	CVD - Blood Pressure	2008	1	1
Microzide	hydrochlorothiazide	CVD - Blood Pressure	2011	1	0
Midamor	amiloride hcl	CVD - Blood Pressure	2011	1	0
miglitol	miglitol	Diabetes - Non-insulin	2017		0
Minipress	prazosin hcl	CVD - Blood Pressure	2008	1	0
minoxidil	minoxidil	CVD - Blood Pressure	2008	1	0
moexipril	moexipril hcl	CVD - Blood Pressure	2008	1	0
moexipril-hydrochlorothiazide	moexipril/hydrochlorothiazide	CVD - Blood Pressure	2008	1	0
molindone	molindone hcl	CNS - Psychosis	2017		0
montelukast	montelukast sodium	Respiratory - Asthma/COPD	2013		0
mycophenolate	mycophenolate mofetil	Immunosuppression	2011	1	0
mycophenolic acid	mycophenolate sodium	Immunosuppression	2014	1	0
Myfortic	mycophenolate sodium	Immunosuppression	2011	1	0
nadolol	nadolol	CVD - Blood Pressure	2008	1	0
nadolol-bendroflumethazide	nadolol/bendroflumethiazide	CVD - Blood Pressure	2008	1	0
nateglinide	nateglinide	Diabetes - Non-insulin	2011		0
Neoral	cyclosporine, modified	Immunosuppression	2011	1	0
Nesina	alogliptin benzoate	Diabetes - Non-insulin	2014		0
nevirapine	nevirapine	Anti-Infectives - HIV/AIDS	2013		0
nevirapine extended-release	nevirapine	Anti-Infectives - HIV/AIDS	2013		1
niacin extended-release	niacin	CVD - Lipid Lowering	2008	1	0
Niacor	niacin	CVD - Lipid Lowering	2010	1	0

© 2024 Wharam JF et al. *JAMA Health Forum.*

eTable 2. Medications on the 2017 Expanded PDL by Therapeutic Category

Drug Listed[1]	Generic Equivalent	Therapeutic Category	First Listed	On Core List	Excludable[2]
Niaspan	niacin	CVD - Lipid Lowering	2008	1	0
nicardipine	nicardipine hcl	CVD - Blood Pressure	2008	1	0
nifedipine	nifedipine	CVD - Blood Pressure	2008	1	0
nifedipine ER	nifedipine	CVD - Blood Pressure	2008	1	0
nimodipine	nimodipine	CVD - Blood Pressure	2010	1	0
nisoldipine	nisoldipine	CVD - Blood Pressure	2010	1	0
Norvasc	amlodipine besylate	CVD - Blood Pressure	2008	1	0
Norvir	ritonavir	Anti-Infectives - HIV/AIDS	2011		0
Novolin 70/30	insulin nph hum/reg insulin hm	Diabetes - Insulin	2008		0
Novolin N	insulin nph human isophane	Diabetes - Insulin	2008		0
Novolin R	insulin regular, human	Diabetes - Insulin	2008		0
Novolog	insulin aspart	Diabetes - Insulin	2008		0
Novolog Mix 70/30	insulin aspart prot/insuln asp	Diabetes - Insulin	2009		0
Odefsey	emtricitab/rilpiviri/tenof ala	Anti-Infectives - HIV/AIDS	2017		0
olanzapine	olanzapine	CNS - Psychosis	2013		0
omega-3 acid ethyl esters	omega-3 acid ethyl esters	CVD - Lipid Lowering	2015	1	0
OneTouch Diabetic Meters	blood-glucose meter	Diabetes - Supplies	2017		0
OneTouch Diabetic Test Strips	onetouch diabetic test strips	Diabetes - Supplies	2017		0
Onglyza	saxagliptin hcl	Diabetes - Non-insulin	2010		0
Oseni	alogliptin benz/pioglitazone	Diabetes - Non-insulin	2014		0
paliperidone ER	paliperidone	CNS - Psychosis	2017		0
pediatric flouride preparations	pediatric flouride preparations	Prenatal & Pediatric	2011	1	0
Perforomist	formoterol fumarate	Respiratory - Asthma/COPD	2008		0
perindopril	perindopril erbumine	CVD - Blood Pressure	2011	1	0
perphenazine	perphenazine	CNS - Psychosis	2008		0
Persantine	dipyridamole	CVD - Clotting/Platelet Therapy	2010	1	0
pindolol	pindolol	CVD - Blood Pressure	2008	1	0
pioglitazone	pioglitazone hcl	Diabetes - Non-insulin	2014		0
pioglitazone-glimepiride	pioglitazone hcl/glimepiride	Diabetes - Non-insulin	2014		0
pioglitazone-metformin	pioglitazone hcl/metformin hcl	Diabetes - Non-insulin	2014		0
Plavix	clopidogrel bisulfate	CVD - Clotting/Platelet Therapy	2008	1	1
Plegridy	peginterferon beta-1a	CNS - Multiple Sclerosis	2017		0
Pletal	cilostazol	CVD - Clotting/Platelet Therapy	2011	1	0
Pradaxa	dabigatran etexilate mesylate	CVD - Clotting/Platelet Therapy	2012	1	0
Prandimet	repaglinide/metformin hcl	Diabetes - Non-insulin	2011		0
Prandin	repaglinide	Diabetes - Non-insulin	2008		0
Pravachol	pravastatin sodium	CVD - Lipid Lowering	2008	1	0
pravastatin	pravastatin sodium	CVD - Lipid Lowering	2008	1	0
prazosin	prazosin hcl	CVD - Blood Pressure	2008	1	0
Precose	acarbose	Diabetes - Non-insulin	2008		0
prenatal vitamins	prenatal vitamins	Prenatal & Pediatric	2008	1	0
Prestalia	perindopril arg/amlodipine bes	CVD - Blood Pressure	2017	1	1
Prevalite	cholestyramine/aspartame	CVD - Lipid Lowering	2008	1	0
Prezcobix	darunavir/cobicistat	Anti-Infectives - HIV/AIDS	2015		1
Prezista	darunavir ethanolate	Anti-Infectives - HIV/AIDS	2011		0
Prinivil	lisinopril	CVD - Blood Pressure	2008	1	0
Proair HFA	albuterol sulfate	Respiratory - Asthma/COPD	2008		0
Proair Respiclick	albuterol sulfate	Respiratory - Asthma/COPD	2016		1
Procardia	nifedipine	CVD - Blood Pressure	2011	1	0

© 2024 Wharam JF et al. *JAMA Health Forum.*

eTable 2. Medications on the 2017 Expanded PDL by Therapeutic Category

Drug Listed[1]	Generic Equivalent	Therapeutic Category	First Listed	On Core List	Excludable[2]
Procardia XL	nifedipine	CVD - Blood Pressure	2008	1	0
Prograf	tacrolimus	Immunosuppression	2008	1	0
propranolol	propranolol hcl	CVD - Blood Pressure	2008	1	0
propranolol-hydrochlorothiazide	propranolol/hydrochlorothiazid	CVD - Blood Pressure	2008	1	0
Proventil HFA	albuterol sulfate	Respiratory - Asthma/COPD	2008		0
Pulmicort Flexhaler	budesonide	Respiratory - Asthma/COPD	2011		0
Pulmicort/Pulmicort Nebulized Solution	budesonide	Respiratory - Asthma/COPD	2008		0
Qbrelis	lisinopril	CVD - Blood Pressure	2017	1	1
Questran	cholestyramine (with sugar)	CVD - Lipid Lowering	2008	1	0
Questran Light	cholestyramine/aspartame	CVD - Lipid Lowering	2008	1	0
quetiapine	quetiapine fumarate	CNS - Psychosis	2013		0
quinapril	quinapril hcl	CVD - Blood Pressure	2008	1	0
quinapril-hydrochlorothiazide	quinapril/hydrochlorothiazide	CVD - Blood Pressure	2008	1	0
QVAR	beclomethasone dipropionate	Respiratory - Asthma/COPD	2008		0
raloxifene	raloxifene hcl	Osteoporosis, Menopause	2015	1	0
ramipril	ramipril	CVD - Blood Pressure	2008	1	0
Rapamune	sirolimus	Immunosuppression	2011	1	0
Rebif	interferon beta-1a/albumin	CNS - Multiple Sclerosis	2011		0
repaglinide	repaglinide	Diabetes - Non-insulin	2014		0
repaglinide-metformin	repaglinide/metformin hcl	Diabetes - Non-insulin	2017		0
Rescriptor	delavirdine mesylate	Anti-Infectives - HIV/AIDS	2011		0
reserpine	reserpine	CVD - Blood Pressure	2008	1	0
Retrovir	zidovudine	Anti-Infectives - HIV/AIDS	2011		0
Rexulti	brexpiprazole	CNS - Psychosis	2017		0
Reyataz	atazanavir sulfate	Anti-Infectives - HIV/AIDS	2011		0
Riomet	metformin hcl	Diabetes - Non-insulin	2009		0
risedronate	risedronate sodium	Osteoporosis, Menopause	2015	1	0
Risperdal	risperidone	CNS - Psychosis	2008		1
risperidone	risperidone	CNS - Psychosis	2008		0
rosuvastatin	rosuvastatin calcium	CVD - Lipid Lowering	2017	1	0
Sandimmune	cyclosporine	Immunosuppression	2011	1	0
Saphris	asenapine maleate	CNS - Psychosis	2010		0
Savaysa	edoxaban tosylate	CVD - Clotting/Platelet Therapy	2015	1	1
Sectral	acebutolol hcl	CVD - Blood Pressure	2008	1	0
Seebri NeoHaler	glycopyrrolate	Respiratory - Asthma/COPD	2017		0
Selzentry	maraviroc	Anti-Infectives - HIV/AIDS	2011		0
Serevent Diskus	salmeterol xinafoate	Respiratory - Asthma/COPD	2008		0
Seroquel	quetiapine fumarate	CNS - Psychosis	2008		1
Seroquel XR	quetiapine fumarate	CNS - Psychosis	2008		0
simvastatin	simvastatin	CVD - Lipid Lowering	2008	1	0
Singulair	montelukast sodium	Respiratory - Asthma/COPD	2008		1
sirolimus	sirolimus	Immunosuppression	2014	1	0
Soltamox	tamoxifen citrate	Breast Cancer Prevention	2008	1	1
Spiriva HandiHaler	tiotropium bromide	Respiratory - Asthma/COPD	2015		0
Spiriva Respimat	tiotropium bromide	Respiratory - Asthma/COPD	2015		0
spironolactone	spironolactone	CVD - Blood Pressure	2008	1	0
spironolactone-hydrochlorothiazide	spironolact/hydrochlorothiazid	CVD - Blood Pressure	2008	1	0
Starlix	nateglinide	Diabetes - Non-insulin	2008		0
stavudine	stavudine	Anti-Infectives - HIV/AIDS	2011		0
Stiolto Respimat	tiotropium br/olodaterol hcl	Respiratory - Asthma/COPD	2016		1

© 2024 Wharam JF et al. *JAMA Health Forum.*

eTable 2. Medications on the 2017 Expanded PDL by Therapeutic Category

Drug Listed[1]	Generic Equivalent	Therapeutic Category	First Listed	On Core List	Excludable[2]
Stribild	elviteg/cob/emtri/tenofo disop	Anti-Infectives - HIV/AIDS	2014		0
Striverdi Respimat	olodaterol hcl	Respiratory - Asthma/COPD	2015		0
Sular	nisoldipine	CVD - Blood Pressure	2008	1	0
Sustiva	efavirenz	Anti-Infectives - HIV/AIDS	2011		0
Symbicort	budesonide/formoterol fumarate	Respiratory - Asthma/COPD	2008		1
Symlinpen	pramlintide acetate	Diabetes - Non-insulin	2014		0
Synjardy	empagliflozin/metformin hcl	Diabetes - Non-insulin	2017		0
tacrolimus	tacrolimus	Immunosuppression	2011	1	0
tamoxifen	tamoxifen citrate	Breast Cancer Prevention	2008	1	0
Tanzeum	albiglutide	Diabetes - Non-insulin	2015		0
Tarka	trandolapril/verapamil hcl	CVD - Blood Pressure	2008	1	0
Taztia XT	diltiazem hcl	CVD - Blood Pressure	2008	1	0
Tecfidera	dimethyl fumarate	CNS - Multiple Sclerosis	2014		0
Tekturna	aliskiren hemifumarate	CVD - Blood Pressure	2008	1	0
Tekturna HCT	aliskiren/hydrochlorothiazide	CVD - Blood Pressure	2008	1	0
telmisartan	telmisartan	CVD - Blood Pressure	2014	1	0
telmisartan-hydrochlorothiazide	telmisartan/hydrochlorothiazid	CVD - Blood Pressure	2014	1	0
telmisartan-amlodipine	telmisartan/amlodipine	CVD - Blood Pressure	2014	1	1
Tenex	guanfacine hcl	CVD - Blood Pressure	2008	1	0
Tenoretic	atenolol/chlorthalidone	Prenatal & Pediatric	2008	1	1
Tenormin	atenolol	CVD - Blood Pressure	2008	1	1
terazosin	terazosin hcl	CVD - Blood Pressure	2008	1	0
terbutaline	terbutaline sulfate	Respiratory - Asthma/COPD	2008		0
Teveten	eprosartan mesylate	CVD - Blood Pressure	2008	1	0
Teveten HCT	eprosartan/hydrochlorothiazide	CVD - Blood Pressure	2008	1	0
Thalitone	chlorthalidone	CVD - Blood Pressure	2011	1	0
Theo-24	theophylline anhydrous	Respiratory - Asthma/COPD	2009		0
Theochron	theophylline anhydrous	Respiratory - Asthma/COPD	2009		0
theophylline	theophylline anhydrous	Respiratory - Asthma/COPD	2008		0
theophylline/guaifenesin	guaifenesin/theophylline	Respiratory - Asthma/COPD	2008		0
thioridazine	thioridazine hcl	CNS - Psychosis	2008		0
thiothixene	thiothixene hcl	CNS - Psychosis	2008		0
Tiazac	diltiazem hcl	CVD - Blood Pressure	2008	1	0
ticlopidine	ticlopidine hcl	CVD - Clotting/Platelet Therapy	2008	1	0
timolol	timolol maleate	CVD - Blood Pressure	2008	1	0
Tivicay	dolutegravir sodium	Anti-Infectives - HIV/AIDS	2014		0
tolbutamide	tolbutamide	Diabetes - Non-insulin	2008		0
Toprol XL	metoprolol succinate	CVD - Blood Pressure	2008	1	0
torsemide	torsemide	CVD - Blood Pressure	2008	1	0
Toujeo	insulin glargine,hum.rec.anlog	Diabetes - Insulin	2015		1
Tradjenta	linagliptin	Diabetes - Non-insulin	2013		0
Trandate	labetalol hcl	CVD - Blood Pressure	2008	1	0
trandolapril	trandolapril	CVD - Blood Pressure	2008	1	0
trandolapril-verapamil	trandolapril/verapamil hcl	CVD - Blood Pressure	2011	1	0
Tresiba	insulin degludec	Diabetes - Insulin	2017		1
triamterene-hydrochlorothiazide	triamterene/hydrochlorothiazid	CVD - Blood Pressure	2008	1	0
Tribenzor	olmesartan/amlodipin/hcthiazid	CVD - Blood Pressure	2012	1	1
Tricor	fenofibrate	CVD - Lipid Lowering	2008	1	1

© 2024 Wharam JF et al. *JAMA Health Forum.*

eTable 2. Medications on the 2017 Expanded PDL by Therapeutic Category

Drug Listed[1]	Generic Equivalent	Therapeutic Category	First Listed	On Core List	Excludable[2]
trifluoperazine	trifluoperazine hcl	CNS - Psychosis	2008		0
Triglide	fenofibrate nanocrystallized	CVD - Lipid Lowering	2008	1	1
Trilipix	fenofibric acid (choline)	CVD - Lipid Lowering	2010	1	1
Triumeq	abacavir/dolutegravir/lamivudi	Anti-Infectives - HIV/AIDS	2008		0
Trizivir	abacavir/lamivudine/zidovudine	Anti-Infectives - HIV/AIDS	2011		0
Trulicity	dulaglutide	Diabetes - Non-insulin	2015		0
Truvada	emtricitabine/tenofovir (tdf)	Anti-Infectives - HIV/AIDS	2011		0
Tudorza Pressair	aclidinium bromide	Respiratory - Asthma/COPD	2014		0
Twynsta	telmisartan/amlodipine	CVD - Blood Pressure	2011	1	1
Uniretic	moexipril/hydrochlorothiazide	CVD - Blood Pressure	2008	1	0
Univasc	moexipril hcl	CVD - Blood Pressure	2008	1	0
Utibron NeoHaler	indacaterol/glycopyrrolate	Respiratory - Asthma/COPD	2017		0
valsartan	valsartan	CVD - Blood Pressure	2015	1	0
valsartan-hydrochlorothiazide	valsartan/hydrochlorothiazide	CVD - Blood Pressure	2014	1	0
Vascepa	icosapent ethyl	CVD - Lipid Lowering	2014	1	0
Vaseretic	enalapril/hydrochlorothiazide	CVD - Blood Pressure	2008	1	1
Vasotec	enalapril maleate	CVD - Blood Pressure	2008	1	1
Ventolin HFA	albuterol sulfate	Respiratory - Asthma/COPD	2008		0
verapamil	verapamil hcl	CVD - Blood Pressure	2008	1	0
verapamil ER	verapamil hcl	CVD - Blood Pressure	2008	1	0
Verelan	verapamil hcl	CVD - Blood Pressure	2008	1	0
Verelan PM	verapamil hcl	CVD - Blood Pressure	2008	1	0
Versacloz	clozapine	CNS - Psychosis	2014		1
Victoza	liraglutide	Diabetes - Non-insulin	2011		0
Videx	didanosine/sodium citrate	Anti-Infectives - HIV/AIDS	2011		0
Videx EC	didanosine	Anti-Infectives - HIV/AIDS	2011		0
Viracept	nelfinavir mesylate	Anti-Infectives - HIV/AIDS	2011		0
Viramune	nevirapine	Anti-Infectives - HIV/AIDS	2011		1
Viramune XR	nevirapine	Anti-Infectives - HIV/AIDS	2012		1
Viread	tenofovir disoproxil fumarate	Anti-Infectives - HIV/AIDS	2011		0
Vitekta	elvitegravir	Anti-Infectives - HIV/AIDS	2015		1
Vospire ER	albuterol sulfate	Respiratory - Asthma/COPD	2011		0
Vraylar	cariprazine hcl	CNS - Psychosis	2017		0
Vytorin	ezetimibe/simvastatin	CVD - Lipid Lowering	2008	1	0
warfarin	warfarin sodium	CVD - Clotting/Platelet Therapy	2008	1	0
Welchol	colesevelam hcl	CVD - Lipid Lowering	2008	1	0
Xarelto	rivaroxaban	CVD - Clotting/Platelet Therapy	2012	1	0
Xigduo XR	dapagliflozin/metformin hcl	Diabetes - Non-insulin	2015		1
Xopenex HFA	levalbuterol tartrate	Respiratory - Asthma/COPD	2008		0
Xopenex Nebulized Solution	levalbuterol hcl	Respiratory - Asthma/COPD	2010		1
zafirlukast	zafirlukast	Respiratory - Asthma/COPD	2012		0
Zaroxolyn	metolazone	CVD - Blood Pressure	2009	1	0
Zebeta	bisoprolol fumarate	CVD - Blood Pressure	2008	1	0
Zerit	stavudine	Anti-Infectives - HIV/AIDS	2011		0
Zestoretic	lisinopril/hydrochlorothiazide	CVD - Blood Pressure	2008	1	1
Zestril	lisinopril	CVD - Blood Pressure	2008	1	1
Zetia	ezetimibe	CVD - Lipid Lowering	2008	1	0
Ziac	bisoprolol/hydrochlorothiazide	CVD - Blood Pressure	2008	1	0
Ziagen	abacavir sulfate	Anti-Infectives - HIV/AIDS	2011		0
zidovudine	zidovudine	Anti-Infectives - HIV/AIDS	2011		0
Zinbryta	daclizumab	CNS - Multiple Sclerosis	2017		0

© 2024 Wharam JF et al. *JAMA Health Forum.*

eTable 2. Medications on the 2017 Expanded PDL by Therapeutic Category

Drug Listed[1]	Generic Equivalent	Therapeutic Category	First Listed	On Core List	Excludable[2]
ziprasidone	ziprasidone hcl	CNS - Psychosis	2013		0
Zocor	simvastatin	CVD - Lipid Lowering	2008	1	0
Zontivity	vorapaxar sulfate	CVD - Clotting/Platelet Therapy	2015	1	0
Zortress	everolimus	Immunosuppression	2012	1	0
Zyflo	zileuton	Respiratory - Asthma/COPD	2008		0
Zyflo CR	zileuton	Respiratory - Asthma/COPD	2008		0
Zyprexa	olanzapine	CNS - Psychosis	2008		1

[1] Capitalized medications were listed on the PDL by brand name, lower case by generic name

[2] Product can be excluded from the PDL by an employer

© 2024 Wharam JF et al. *JAMA Health Forum.*

B. Imputation of Preventive Drug List benefits

The national insurer represented in our data markets two basic types of Preventive Drug List (PDL): a Core PDL and an Expanded PDL. The Core PDL contains medications in several therapeutic categories (including hypertension, hyperlipidemia, breast cancer prevention, blood clotting/platelet therapy, organ rejection, osteoporosis, prenatal vitamins) that can be offered to members with reduced or no cost sharing as a pharmacy benefit enhancement; the Expanded PDL contains additional therapeutic categories (e.g., diabetes, asthma, HIV/AIDS, multiple sclerosis, psychosis) that can be optionally added to the Core PDL. We did not have access to a variable indicating which accounts offered PDL coverage in each benefit year, so we used member out-of-pocket payment amounts on pharmacy claims to impute the annual presence and type of PDL for each account. The steps in conducting the imputation are outlined in eTable 3.

Our data source contains a unique identifier for each account. Large employers can have multiple simultaneous accounts, for example, if their insurance contracting is organized by state or region. We joined all accounts for individual employers and their anniversary months using information provided by the data vendor and by tracking multiple member switches between account numbers on anniversary dates. We then imputed the presence and type of PDL for employers as a whole.

Although PDLs are somewhat standardized, employers have discretion over which medicines they include, since some products being listed as optional. The numbers of products listed by category on the insurer's 2017 PDLs are shown in eTable 1 and the names of the specific products listed are in eTable 2. For our imputation, we included all products (brand or generic) that were chemically equivalent to medications listed on the Core or Expanded PDL using equivalency data from the First DataBank© National Drug Data File PlusTM (First DataBank, Inc., San Bruno, CA). We had access to copies of the insurer's annual Core and Expanded PDLs for the years 2008-2017 and we varied the products used for the imputation each year; we used the 2008 PDL list for earlier years. We flagged products as potentially excludable in a year if they were listed that way on the annual PDLs. We identified products as being on the PDL Core List only, on the PDL Expanded List (which also includes all products on the Core List), or unlisted (on neither list). We also separately identified products listed in three large therapeutic classes: antihypertensive and antihyperlipidemic products (which together comprise ~75% of the medications on the PDL Core List) and antidiabetics (oral antidiabetics and insulin, which comprise ~33% of the medications that can be added on the PDL Expanded List).

Typically, members pay copays for most medications until an annual out-of-pocket maximum has been met. However, for some health plans, members must pay the full cost of medications until an annual deductible amount has been met, after which they either pay copays or have no out-of-pocket obligation. By IRS regulation, all members in HDHPs linked to health savings accounts (HDHP-HSAs) must pay the full cost of medications until meeting their annual deductible. Minimum annual deductible amounts for HDHP-HSAs are regulated by the IRS; minimum deductibles were $1,300 for individual coverage and $2,600 for family coverage in 2017. However, specific preventive medications, including those on PDLs, can be dispensed without deductible payment. Thus, PDLs are particularly important for members in HDHP-HSAs.

For each account, we first determined the percentage of claims in each benefit month for which members paid a deductible or a copay, requiring a minimum of 3 fills in a category to evaluate a given month. We evaluated percentages of claims with deductible/copay for NDCs on the Core PDL, the Expanded PDL, and unlisted; we also separately evaluated percentages of claims with cost sharing in the three therapeutic groups. If ≤10% of filled claims in an account paid a deductible/copay for products in a category while ≥30% of claims for unlisted medicines had a deductible/copays charged, the month was flagged as a potential PDL month. We evaluated only the first 6 months of each benefit year since increasing percentages of members have no cost sharing for medications later in a benefit year due to having met their deductible or annual out-of-pocket maximum, making it more difficult to reliably compare listed and unlisted products. We next determined for each account the percentages of claims on each list and in each therapeutic group that had deductible/copay payments during the entire benefit year, requiring at least 10 claims in a category to evaluate the year. If all claims in an account had no member cost sharing (i.e., all medicines dispensed free to members), the benefit year was excluded for the account.

© 2024 Wharam JF et al. *JAMA Health Forum.*

We finally assigned each benefit year to a PDL status (Expanded PDL, Core PDL only, no PDL) using rules based on the monthly and annual summaries. Accounts with deductibles for medicines during the benefit year (e.g., all HDHP-HSAs) were evaluated based on deductible amounts on claims, while non-deductible accounts were evaluated based on copay amounts, both overall and for generic and brand products separately. Meeting rules for having no out-of-pocket payments for all three therapeutic classes in a benefit year was considered equivalent to meeting rules for the Expanded PDL, while meeting rules for having no out-of-pocket payments for antihypertensives and antihyperlipidemics but not antidiabetics in a benefit year was considered equivalent to meeting rules for the Core PDL. If an account met rules for both core and expanded PDLs in a benefit year, the Expanded PDL was given precedence.

eTable 3. Steps in Imputing Presence of PDL at the Account Level and Determining Full Replacement of No PDL With PDL at Linked Account Level

PDL status of products<br>• Identify medicines listed on Core and Expanded PDLs in each calendar year, as well as those in three large therapeutic classes (antihypertensives, antihyperlipidemics, antidiabetics)<br>• Identify National Drug Codes (NDCs) for all equivalent products containing the medicines listed<br>• Identify products listed as optionally excludable (default imputation excludes these from analysis)
Monthly PDL status for each account based on monthly fills<br>• Determine percentages of monthly claims with deductible/copay (≥3 fills to evaluate) for NDCs on the Expanded PDL, Core PDL, Unlisted, and the three large therapeutic classes<br>• If ≤10% of claims for NDCs on Core/Expanded PDLs or in therapeutic classes have deductible/copay and ≥30% of unlisted medicines have deductible/copay, then month is flagged as a potential PDL month<br>• Evaluate only first six months of the benefit year since an increasing percentage of individuals with medication deductibles exceed their required deductible each month and have no cost sharing thereafter
Annual PDL status for each account based on annual fills<br>• Count and determine percentages of annual claims with deductible/copay (minimum 10 fills to evaluate) for NDCs on the Core/Expanded PDLs, unlisted NDCs, and the three therapeutic categories<br>• If all fills in an account (both Core/Expanded PDL and unlisted) have $0 deductibles and $0 copays (i.e., no member cost sharing), then account is excluded<br>• Accounts with evidence of deductibles in a benefit year evaluated based on deductible amounts on claims, while copay only accounts evaluated based on copay amounts<br>• Benefit year is flagged as Core/Expanded PDL if it meets any of the following conditions:<br>o At least 5 of the first 6 months in year met rule for potential Core/Expanded PDL month above<br>o ≤10% of fills for Core/Expanded PDL NDCs in year had deductible/copay >$0 and ≥30% of fills for unlisted medicines had deductible/copay >$0<br>o No fills for Core/Expanded PDL NDCs in year had deductible/copay >$0 and ≥5 fills for unlisted medicines had deductible/copay >$0

© 2024 Wharam JF et al. *JAMA Health Forum.*

- No fills for generic Core/Expanded PDL NDCs in year had copay >$0 and ≥5 fills for unlisted generic medicines had copay >$0<br>- No fills for brand Core/Expanded PDL NDCs in year had copay >$0 and ≥5 fills for unlisted brand medicines had copay >$0<br>• Benefit year is considered Expanded PDL if account meets rules for both Expanded PDL and Core PDL
Annual percentage coverage with PDL and full replacement at employer level.<br>• Link individual accounts for an employer based on a variable supplied by the vendor or if there is strong evidence of multiple members switching back and forth between accounts on anniversary dates<br>• For linked employer accounts:<br>- If >85% of members in an employer have evidence of a PDL in a benefit year, the employer is determined to uniformly offer PDL coverage in that year; if >85% of members have no evidence of a PDL, then the employer is uniformly assigned to no PDL status<br>- Employers that transition from <15% of members with a PDL in one benefit year to >85% of members with a PDL in the following benefit year are considered to have fully replaced no PDL coverage with PDL coverage

A benefit year was determined to be an Expanded PDL year for an account if it met imputation rules based on: (1) number of imputed PDL months in the first 6 months of a benefit year; or (2) difference in percentage or number of Core/Expanded PDL fills vs, unlisted fills in a year having $0 deductible/copay, either overall or for generic and brand products separately.

In this study, we restrict analysis to employers that offer a single PDL benefit design to members (i.e., PDL or no PDL). To determine employer PDL status, we link all contemporaneous accounts of each employer using a linking variable supplied by the data vendor which we supplemented by analysis of multiple members transitioning between accounts in anniversary months (see Appendix Section 3). We use a common industry standard that defined situations in which >85% of members in linked accounts have the same benefit design as uniformly offering that design. If ≥15% to ≤85% of enrollees at the employer had access to a PDL in a given year, we considered that the employer offered enrollees a choice to have a PDL benefit or not, and we excluded employers and their members in these benefit years from the study. To determine employers that switched to a PDL benefit design, we used the linked account PDL/non-PDL status to identify situations in which an employer replaced a full year of no PDL coverage with a consecutive full year with PDL coverage. We identified a control group of members working for employers that continued all members without PDL coverage for two consecutive full years; if employers had more than one such period without PDLs, one two-year period was randomly selected to be in the control pool.

C. Identifying patients with diabetes

We identified all patients aged 12 to 64 diagnosed with diabetes. Using explicit criteria and codes described for constructing the Comprehensive Diabetes Care measures in HEDIS (Healthcare Effectiveness Data and Information Set), we identified patients with diabetes as those who had diagnosis codes indicating diabetes (eTable 4) in medical service claims (1 inpatient claim or 2 outpatient claims within one year), who had a hospitalization for a diabetes DRG (Diagnosis Related Group), or who had pharmacy claims for insulin or a non-insulin antidiabetic medication other than metformin alone (excluded because it can also be used to treat prediabetes or polycystic ovary syndrome). The date of first diabetes diagnosis or diabetes medication dispensing was required to precede the index month.

© 2024 Wharam JF et al. *JAMA Health Forum.*

eTable 4. Diagnosis and Medication Codes Used to Define Patients With Diabetes

**Code Description**	**Code type**	**Code**
Include if:		
Diagnosis of diabetes mellitus	ICD-9	250.0-250.93, 357.2, 362.0, 366.41
	ICD-10	E10.xxx-E11.xxx
Diagnosis of diabetes in pregnancy and childbirth	ICD-9	648.xx
	ICD-10	O24.0xx, O24.1xx, O24.3xx, O24.8xx
Hospitalization for diabetes[1]	DRG[1]	294, 295
	MS-DRG[2]	637, 638, 639
Use of oral antidiabetic medications[3]	AHFS	68200200, 68200300, 68200500, 68200600, 68201600, 68201800, 68202000, 68202800, 68209200
Use of insulin	AHFS	682008xx
Exclude if:		
Polycystic ovary syndrome	ICD-9	256.4
Other disorders of pancreatic internal secretion	ICD-9	251.8
Poisoning by adrenal cortical steroid	ICD-9	962.0
Diabetes due to underlying condition	ICD-10	E08.xxx
Drug or chemical induced diabetes mellitus	ICD-10	E09.xxx
Gestational diabetes mellitus	ICD-9	648.8x
	ICD-10	O24.4xx

[1]Used before 10/1/2007; [2]Used on or after 10/1/2007; [3] 68200400 (biguanides) alone not sufficient for diagnosis

Abbreviations: ICD-9, International Classification of Diseases, 9th Revision, Clinical Modification; ICD-10-CM, International Classification of Diseases, 9th Revision, Clinical Modification; DRG, Diagnosis Related Group; AHFS, American Hospital Formulary Service Classification

D. Description of variables used to match or stratify analyses

i. Person-level variables: We classified number baseline acute, preventable complication days per member as 0, 1, 2-3, 4-7, 8-21, 22-60, and 60+. We used version 11.1 of the Johns Hopkins ACG® System[1,2] to calculate members' baseline period morbidity score. The algorithm uses age, gender, and ICD-9-CM codes to calculate a morbidity score and the average of the reference population is 1.0.[2] Researchers have validated the index against premature mortality.[1] We calculated quartile of ACG score among the baseline intervention group and control pool population. To derive proxy demographic measures, the data vendor linked members' most recent residential street addresses to their 2010 US Census tract.[3] Census-based measures of socioeconomic status have been validated [4,5] and used in multiple studies to examine the impact of policy changes on disadvantaged populations.[6-8] Using 2010-2014 American Community Survey[9] census tract-level data and validated cut-points,[4,5] we created categories that defined residence in neighborhoods with below-poverty levels of <5%, 5%-9.9%, 10%-19.9%, and ≥20%. We classified members as from predominantly white, black, or Hispanic neighborhoods if they lived in a census tract with at least 75% of members of the respective race/ethnicity. We then applied a superseding ethnicity assignment using flags provided by the data vendor based on analyzing full names and geographic locations of individuals. We classified remaining members as from mixed race/ethnicity neighborhoods. This validated approach of combining surname analysis and census data has positive and negative predictive values of approximately 80 and 90 percent, respectively.[10] Using the above categories for cardiometabolic medications (eTable 2), we classified member baseline quarter of first fill for non-insulin antidiabetic, insulin, antihypertensive, and lipid lowering agent, if any. Other variables included age as a category (12-17, 18-29, 30-44, 45-49, 50-54, 55-59, and 60-64 years); sex; US region (West, Midwest, South, Northeast); baseline out-of-pocket spending decile; decile of total baseline total standard costs.

We also classified members by their baseline and follow-up clinician network "narrowness" (e.g., narrow = HMO; broad = PPO) and deductible level. Deductible levels included $0-500, $1000-2499, ≥$2500, choice,

© 2024 Wharam JF et al. *JAMA Health Forum.*

and other. Our algorithm for imputing employer-mandated membership in high deductible health plans is described below.

To determine employer deductible levels, we used a benefits type variable that we had for most smaller employers (with approximately 100 or fewer employees). For larger accounts, we took advantage of the fact that health insurance claims data are the most accurate source for assessing out-of-pocket obligations among patients who utilize health services. Our claims data contained an in-network/ out-of-network individual deductible payment field. For patients who use expensive or frequent services, the sum of their yearly deductible payments adds up to clearly identifiable exact amounts such as $500.00, $1000.00, $2000.00, etc. When even several members have these same amounts, it provides strong evidence that the employer offered such an annual deductible level. It is also possible to detect employers or accounts that offer choices of deductible levels when multiple employees have deductibles at two or more levels, such as 20 employees with an exact annual amount of $1000.00 and 12 employees with $500.00. For employer accounts with at least 10 enrollees, we therefore summed each member's in-network (individual-level) deductible payments and number of claims over the enrollment year and assessed other key characteristics such as percentage with Health Savings Accounts. We randomly selected half of the employer account data set that contained both our calculated account characteristics (independent variables, below) and actual annual deductible levels from the benefits table (dependent variable, after categorization; below). We then used a multinomial logistic model that predicted the 4-level outcome of individual-level deductible ≤$500/$500-$999/$1000-$2499/≥$2500 (again, dependent variable) based on multiple aggregate account characteristics (independent variables) comprising calendar year, the percentage with Health Savings Accounts and Health Reimbursement Arrangements, the category of deductible payment amount per account ($0 to <$100, ≥$100 to ≤$500, >$500 to <$1000, ≥$1000 to <$2500, ≥$2500) in the 75th percentile of payments, the percentage of employees reaching exact deductible levels or with deductible payments but not reaching an exact deductible level, the percentage of employees who never use any services, the employer account size, and the percentage of enrollees per account with summed whole dollar annual deductible amounts (from claims data) between $0 to <$100, ≥$100 to ≤$500, >$500 to <$1000, ≥$1000 to <$2500, ≥$2500.

This predictive model output the probability that accounts had deductibles in the four categories (summing to 1.0) and we assigned the account to the level that had the highest probability. We overwrote this assignment with the most common whole number deductible amount per year if it was not zero, and with the second most common whole number deductible amount if the most common amount was zero and at least 10 members had the value of the second most common whole number deductible amount. If an account had members with both enrollment and evidence of utilization, but never had any amounts in the deductible field, we assigned that account to the <$500 deductible level. If an account had only members that reached a whole number annual deductible amount such as $1000.00 or $2000.00, we assigned the most common deductible amount as the account's deductible if that amount was greater than or equal to $1000 and to the 95% percentile value if that number was less than $1000. If 100% of employees had Health Savings Accounts, we also overwrote any previous assignment to classify the account as a high-deductible account. We assigned accounts to have a choice between deductible levels of $1000 to $2499 and ≥$2500 when both were common and one accounted for at least 85% of $1000-$2499 or ≥$2500 deductible levels reached per account. If we detected accounts that had sufficient enrollees with whole number deductible levels both above and below $1000 (e.g. $250.00 and $1500.00), we assigned the accounts' category as "choice," applying a similar 85% rule. Finally, for any account that had gold standard deductible level information in our benefits file, we overwrote any previous imputed deductible level.

To validate our imputation algorithm, we took advantage of the fact that we have exact deductible amounts for some larger accounts. Using the other half of the split sample, we tested the sensitivity and specificity of our imputation. After excluding accounts classified as having a choice of high or low deductible level, we found sensitivity of 98.0% and specificity of 96.1% for identifying HDHP accounts. We then used an employer ID and an algorithm that determined linked employer subaccounts to identify and classify employers' subaccounts per benefit year and thus whether employers offered deductible levels of to $0 to ≤$500, >$500 to <$1000, ≥$1000 to <$2500, ≥$2500, choice of ≥$1000 to <$2500 or ≥$2500, and choice of deductible level <$1000 or ≥$1000. We classified employers with deductible levels of $0 to ≤$500 as low-deductible plans and those with

© 2024 Wharam JF et al. *JAMA Health Forum.*

deductible levels of ≥$1000 to <$2500, ≥$2500, or choice of ≥$1000 to <$2500 or ≥$2500 as high-deductible plans.

ii. Employer-level variables: These included employer size category (10-99, 100-999, 1000-4999, ≥5000 enrollees); calendar quarter of the index date; calendar year period of the index date (2004-2007, 2008-2009, 2010-2011, 2012, 2013, 2014, 2015, 2016); baseline proportion of members in age category (0-19, 20-29, 30-39, 40-49, 50-64, and 65+ years); proportion of female members; proportion of members in census tract poverty category (below-poverty levels of <5%, 5%-9.9%, 10%-19.9%, and ≥20%); baseline decile of annual employer medical and pharmacy standardized costs and out-of-pocket costs per member per month; baseline mean ACG morbidity score among members with 12 months of baseline enrollment.

E. Assignment of index dates to control group members

Among control pool employers with only 2 benefit years of enrollment, we assigned the index date at the employer's midpoint anniversary date (date when health insurance benefits can change year-on-year). For control pool employers with more than one eligible index date, we randomly assigned an anniversary date as the index date with weighting to ensure a similar ratio of intervention to control members over calendar time.

F. Measures

i. Intermediate measures: We created intermediate measures of antidiabetic medication use to determine if the PDL meaningfully improved use. We assessed these measures during the middle 8-months of the baseline (months 3-10) and follow-up period (months 15-22) to avoid measurement of carryover 90-day fills and anticipatory utilization increases that can occur when health plan members are aware of an upcoming change in benefit generosity.

30-day equivalent non-insulin antidiabetic medication fills and insulin fills: Using the First DataBank National Drug Data File Plus™ (First DataBank, Inc., San Bruno, CA), we captured all pharmacy claims for agents classified as non-insulin antidiabetic agents and insulin. We used the days supply field on the claim to generate 30-day equivalent fills. For example, we would classify a claim for 90 days of metformin as three 30-day equivalent metformin fills. We assessed the number of 30-day equivalent non-insulin antidiabetic fills and insulin fills per member per study period then calculated the mean number per person in the study groups.

Higher use of antidiabetic agents: We again used 30-day antidiabetic medication fill equivalents per person and divided by months of enrollment per study period. We flagged members who had a mean of at least 1.0 fills per period as having "higher" use.

ii. Primary measure

Acute, preventable diabetes complication days: We sought to create a measure of telehealth, outpatient, observation stay, emergency department, and hospital visits indicating that a patient had experienced a diabetes complication that can be caused by deferring or skipping necessary care.

Overview: We defined acute, preventable diabetes complications as symptoms or conditions (when coded by clinicians as the primary diagnosis) that: (1) could be associated with delaying recommended or urgent diabetes-related outpatient or emergency department (ED) care (including prescription drug use) for up to 4 months, (2) must self-present from home to the outpatient or ED setting, (3) require timely care by medical professionals, and (4) can cause serious morbidity or mortality if left untreated. The eFigure displays the 5-step decision algorithm we used to operationalize this definition on claims-data-based diagnoses. Our general goal was to choose specific primary diagnoses with a high degree of face validity for being potentially related to deferral of appropriate care. Other important considerations were that complications must self-present (i.e., asymptomatic conditions only detectable by clinicians were excluded), that the conditions require urgent or emergent care by a medical professional (i.e., they could not be treated at home), and that certain diagnoses have different meanings when coded in the outpatient or high-acuity setting. That is, diagnoses might represent a complication in the high-acuity setting but not the outpatient setting. For example, we coded

© 2024 Wharam JF et al. *JAMA Health Forum.*

“congestive heart failure” as an acute complication only if coded in the ED or hospital, given that office visits for this condition often represent routine monitoring rather than complications.

© 2024 Wharam JF et al. *JAMA Health Forum.*

eFigure. Decision Algorithm for Classifying the Primary *ICD* Diagnosis at Outpatient and High-Acuity Visits as Complications of Diabetes Caused by Short-Term Reduced Care Access, Emphasizing Specificity

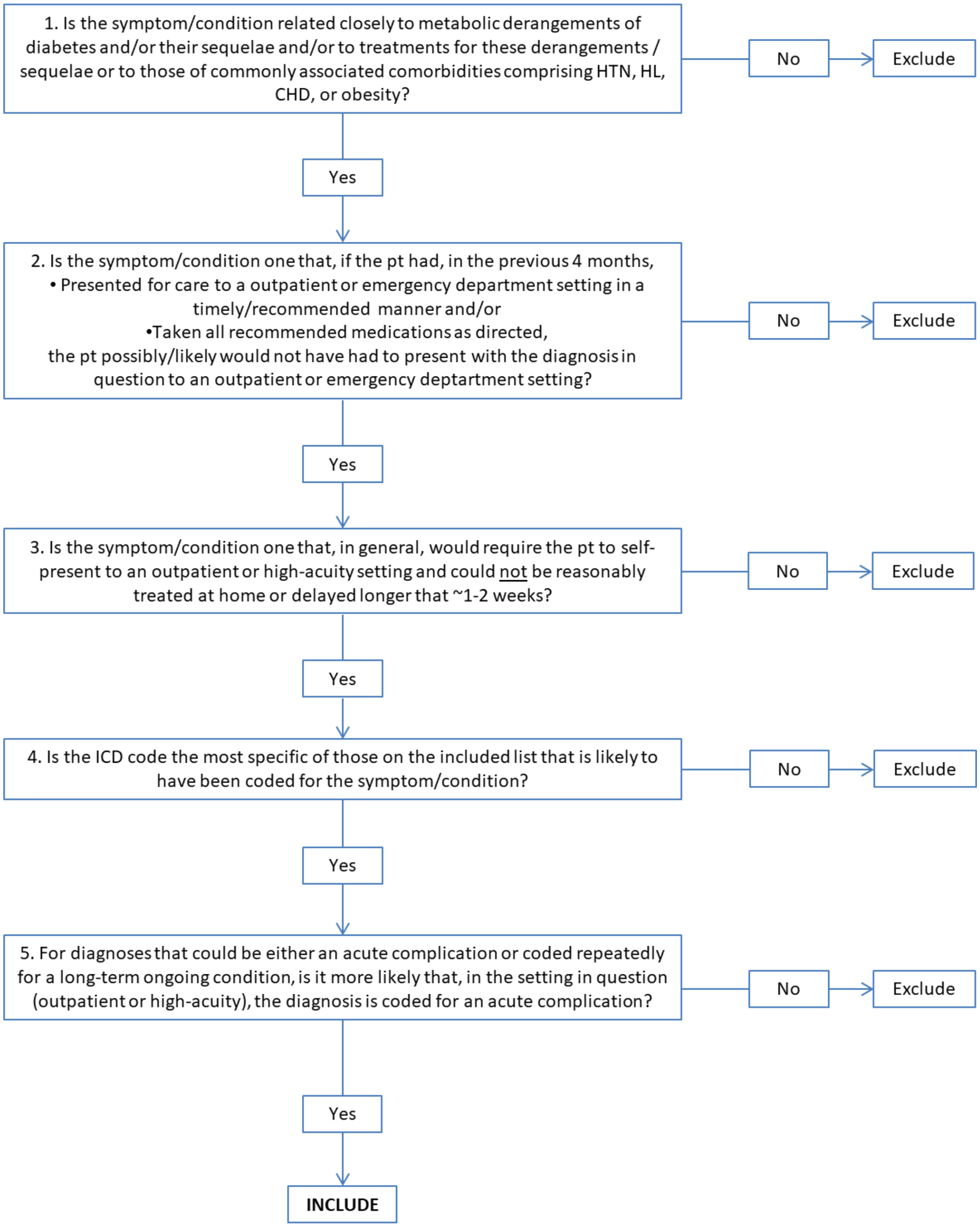

Candidate list of ICD codes: For an earlier study,[11] we developed an initial set of candidate ICD-9 codes using a published list of acute and long-term diabetes complications used to examine the economic impacts of diabetes.[12] We also included diagnoses from the Agency for Healthcare Research and Quality's claims-based Prevention Quality Indicators (http://www.qualityindicators.ahrq.gov/ Modules/pqi_resources.aspx) diabetes complications measures which include a smaller number of acute and long-term diabetes complications. Two

© 2024 Wharam JF et al. *JAMA Health Forum.*

clinicians on the study team then added three diagnoses with a high degree of face validity for being potentially related to gaps in appropriate diabetes and diabetes-related care: hypoglycemia, influenza, and pneumonia.

Classification for inclusion in final measure: For the initial study,[11] two clinicians then independently applied a 5-step decision algorithm similar to that shown in eFigure to each of the ICD-9 codes, separately classifying them as a potential acute complication when coded as the *primary* diagnosis in either the outpatient or high-acuity setting. We excluded any diagnoses (the "No" branches as shown in eFigure) for which the answer to any of the 5 steps was no or was uncertain or ambiguous, in order to prioritize specificity over a sensitivity. After each clinician classified the diagnoses independently, we selected for inclusion in the final measure only those ICD-9 codes for which both clinicians agreed that the diagnosis represented a recent acute complication. This generated a list of 64 outpatient and 89 emergency department acute diabetes complication ICD-9 codes. We then validated this measure by determining that outpatient and ED visits with these complication diagnoses were associated with higher odds (4.10 [3.98, 4.23] and 3.02 [2.96, 3.08]), respectively) of subsequent hospitalization compared with other types of outpatient or ED visits.[11]

For the current study, we updated the measure to ICD-10 codes by using the General Equivalence Mappings approach.[13] This approach created a list of candidate ICD-10 codes as well as a smaller number of ICD-9 codes added to the original list. An internal medicine clinician (J.F.W.) then re-applied the above 5-step criteria to each candidate code to flag those that represented acute, preventable diabetes complications in either the outpatient (telehealth or office visit) or high-acuity (ED, observation stay, or inpatient) setting. 3094 distinct ICD codes were included. The internal medicine clinician then assigned each code into one of 15 clinical categories and 20 clinical subcategories. The clinical categories comprised: Abnormal blood glucose or closely related metabolic abnormalities, Acute Kidney Failure, Cardiac arrest, Cardiac arrythmias and conduction disorders, Congestive heart failure or closely related abnormalities, Diabetic ulcer, Glaucoma, Hyperkalemia, Hypertensive emergency, Hypotension or shock, Infections or closely related conditions, Myocardial infarction or transient coronary artery ischemia, Neuropathy, Non-cardiac, non-cerebral artery complications, and Stroke or transient neurological deficit. eTable 5 displays the frequency of acute, preventable diabetes complication days by clinical subcategory at baseline. Infections were the most common complications type.

© 2024 Wharam JF et al. *JAMA Health Forum.*

eTable 5. Ranked Frequency of Categories of Acute, Preventable Complication Days at Baseline

Rank	Acute Complication Category	% of total	Cumulative %
1	Cellulitis, abscess, and related infections	26.14	26.14
2	Urinary tract infections	18.61	44.75
3	Serious respiratory infections	10.00	54.75
4	Stroke or transient neurological deficit	7.24	61.99
5	Myocardial infarction or transient coronary artery ischemia	7.17	69.16
6	Abnormal blood glucose or closely related metabolic abnormalities	6.43	75.59
7	Other infections	5.59	81.18
8	Otitis externa	5.18	86.36
9	Bacterial upper respiratory tract infections	3.38	89.74
10	Acute kidney failure	2.69	92.43
11	Congestive heart failure or closely related abnormalities	2.29	94.72
12	Cardiac arrythmias and conduction disorders	2.21	96.93
13	Hypotension or shock	1.24	98.17
14	Hyperkalemia	0.66	98.83
15	Non-cardiac, non-cerebral artery complications	0.59	99.42
16	Diabetic ulcer	0.32	99.74
17	Cardiac arrest	0.12	99.86
18	Hypertensive emergency	0.08	99.94
19	Glaucoma	0.03	99.97
20	Neuropathy	0.02	99.99

Creation of acute diabetes complication visit measure*:* Using claims data for our populations of interest, we then captured the primary diagnosis coded by clinicians (using evaluation and management CPT codes and the associated first ICD codes) for any visit in the outpatient (telehealth, office) or high-acuity (observation, emergency department, hospital) setting. We used separate primary diagnosis lists for outpatient and high-acuity settings given the context-specific nature of diagnoses noted above.

Creation of acute, preventable diabetes complication episodes and associated days: We created acute diabetes complication episodes by generating 10-day windows (beginning with the day of an acute complication visit to the outpatient or high-acuity setting) during which we captured visits that had the same cluster or relevant diagnoses (e.g., all pneumonia diagnosis codes). This approach prevented "double counting" of situations such as a 3-day hospitalization for pneumonia followed by a related emergency department visit 2 days later. Our measure of interest was the number of days presenting to the health system for the complication that arose within the 10-day episode. For example, an emergency department visit for diabetic ketoacidosis on day 1 followed by a 3-day hospitalization would represent 4 acute complication days. We chose this measure because days "lost" to, or affected by, preventable health care utilization are important both to patients and employers. We assigned 1 complication day per telehealth or office visit. We assigned the full duration of ED visits and observation stays that began during the 10-day episode but winsorized at 3 days. Similarly, we included each hospitalization day and winsorized at 7 days to minimize the influence of outliers. Complication days were generally confined to the 10-day window but, in the case of ED visits, observation stays, and hospitalizations, could extend beyond the 10-day period. We did not double count days. For example, if a telehealth, outpatient, and ED visit happened on the same day, this only counted as a single complication day.

II. Results

A. Test of baseline trend differences

We tested for baseline trend differences in our primary outcome using linear regression analysis with clustering at the person level.[14] We modeled the continuous outcome of cumulative complication days per month of the

© 2024 Wharam JF et al. *JAMA Health Forum.*

baseline year. The term of interest was an interaction between study group (PDL versus control) and month (1-12) and we adjusted models using weights from the match. The study group by month term indicated whether the intervention and control groups had divergent baseline trends in acute, preventable complication days. We ran these segmented regression models for the overall group and the lower- and higher-income subgroups. Magnitudes of monthly trend differences were extremely small across these groups and none were statistically significant (eTable 6).

eTable 6. Tests of Baseline Monthly Trend Differences in Acute, Preventable Complication Days[1] Between PDL and Control Groups

Group	Baseline trend difference, days per month	Robust standard error	t Value	p Value
Overall	0.0004302	0.0010015	0.43	0.668
Higher-income[2]	0.0003086	0.0011877	0.26	0.795
Lower-income[3]	0.0005378	0.0015706	0.34	0.732

Abbreviations: PDL, preventive drug list. [1]Includes visits to the outpatient, telehealth, emergency department, observation, and inpatient settings for conditions such as bacterial infections, hyperglycemia-related conditions, and acute vascular events. [2]Residence in census tracts with fewer than 10% of households below the federal poverty level based on 2010-2014 American Community Survey. [3]Residence in census tracts with 10% or more of households below the federal poverty level based on 2010-2014 American Community Survey.

© 2024 Wharam JF et al. *JAMA Health Forum.*

eReferences

1. Reid RJ, Roos NP, MacWilliam L, Frohlich N, Black C. Assessing population health care need using a claims-based ACG morbidity measure: a validation analysis in the Province of Manitoba. *Health services research*. Oct 2002;37(5):1345-64.

2. ACG. The Johns Hopkins ACG System. Accessed 2 June, 2018. https://www.hopkinsacg.org/advantage/

3. USCensus. Census 2000 Gateway. U.S. Bureau of the Census. Accessed 2 June, 2018. https://www.census.gov/main/www/cen2000.html

4. Krieger N. Overcoming the absence of socioeconomic data in medical records: validation and application of a census-based methodology. *American Journal of Public Health*. 1992;82(5):703-10.

5. Krieger N, Chen JT, Waterman PD, Rehkopf DH, Subramanian SV. Race/ethnicity, gender, and monitoring socioeconomic gradients in health: a comparison of area-based socioeconomic measures--the public health disparities geocoding project. *American Journal of Public Health*. 2003;93(10):1655-71.

6. Trivedi AN, Zaslavsky AM, Schneider EC, Ayanian JZ. Relationship between quality of care and racial disparities in Medicare health plans. *Jama*. Oct 25 2006;296(16):1998-2004.

7. Trivedi AN, Rakowski W, Ayanian JZ. Effect of cost sharing on screening mammography in medicare health plans. *The New England journal of medicine*. Jan 24 2008;358(4):375-83.

8. Selby JV, Fireman BH, Swain BE. Effect of a copayment on use of the emergency department in a health maintenance organization. *New England Journal of Medicine*. 1996;334(10):635-41.

9. American Community Survey. Accessed 28 September, 2017. https://www.census.gov/programs-surveys/acs/

10. Fiscella K, Fremont AM. Use of geocoding and surname analysis to estimate race and ethnicity. *Health services research*. Aug 2006;41(4 Pt 1):1482-500.

11. Wharam JF, Zhang F, Eggleston EM, Lu CY, Soumerai S, Ross-Degnan D. Diabetes Outpatient Care and Acute Complications Before and After High-Deductible Insurance Enrollment: A Natural Experiment for Translation in Diabetes (NEXT-D) Study. *JAMA Intern Med*. Jan 09 2017;doi:10.1001/jamainternmed.2016.8411

12. American Diabetes A. Economic costs of diabetes in the U.S. in 2012. *Diabetes care*. Apr 2013;36(4):1033-46. doi:10.2337/dc12-2625

13. Butler R. The ICD-10 General Equivalence Mappings. Bridging the translation gap from ICD-9. *J AHIMA*. Oct 2007;78(9):84-5.

14. Wagner AK, Soumerai SB, Zhang F, Ross-Degnan D. Segmented regression analysis of interrupted time series studies in medication use research. *Journal of clinical pharmacy and therapeutics*. Aug 2002;27(4):299-309.

© 2024 Wharam JF et al. *JAMA Health Forum.*
