# Supplementary material for: Acute Diabetes Complications After Transition to a Value-Based Medication Benefit
Source: JAMA Health Forum. 2024 Feb 9;5(2):e235309. doi: 10.1001/jamahealthforum.2023.5309 (PMC10858396; doi:10.1001/jamahealthforum.2023.5309)
Supplement: Supplement 2. — Data Sharing Statement [file jamahealthforum-e235309-s002.pdf]

Data Sharing Statement

Wharam. Acute Diabetes Complications After Transition to a Value-Based Medication Benefit. *JAMA Health Forum*. Published February 09, 2024. doi:10.1001/jamahealthforum.2023.5309

Data
Data available: No

Additional Information
Explanation for why data not available: The data vendor does not allow access to their data without a data use agreement.
